# Current Treatment Options and Therapeutic Insights for Gastrointestinal Dysmotility and Functional Gastrointestinal Disorders

**DOI:** 10.3389/fphar.2022.808195

**Published:** 2022-01-25

**Authors:** Rajan Singh, Hannah Zogg, Uday C Ghoshal, Seungil Ro

**Affiliations:** ^1^ Department of Physiology and Cell Biology, Reno School of Medicine, University of Nevada, Reno, NV, United States; ^2^ Department of Gastroenterology, Sanjay Gandhi Postgraduate Institute of Medical Sciences, Lucknow, India

**Keywords:** gastroparesis, functional dyspepsia, irritable bowel syndrome, visceral hypersensitivity, impaired barrier function, prucalopride, relamorelin, fecal microbiota transplantation

## Abstract

Functional gastrointestinal disorders (FGIDs) have been re-named as disorders of gut-brain interactions. These conditions are not only common in clinical practice, but also in the community. In reference to the Rome IV criteria, the most common FGIDs, include functional dyspepsia (FD) and irritable bowel syndrome (IBS). Additionally, there is substantial overlap of these disorders and other specific gastrointestinal motility disorders, such as gastroparesis. These disorders are heterogeneous and are intertwined with several proposed pathophysiological mechanisms, such as altered gut motility, intestinal barrier dysfunction, gut immune dysfunction, visceral hypersensitivity, altered GI secretion, presence and degree of bile acid malabsorption, microbial dysbiosis, and alterations to the gut-brain axis. The treatment options currently available include lifestyle modifications, dietary and gut microbiota manipulation interventions including fecal microbiota transplantation, prokinetics, antispasmodics, laxatives, and centrally and peripherally acting neuromodulators. However, treatment that targets the pathophysiological mechanisms underlying the symptoms are scanty. Pharmacological agents that are developed based on the cellular and molecular mechanisms underlying pathologies of these disorders might provide the best avenue for future pharmaceutical development. The currently available therapies lack long-term effectiveness and safety for their use to treat motility disorders and FGIDs. Furthermore, the fundamental challenges in treating these disorders should be defined; for instance, 1. Cause and effect cannot be disentangled between symptoms and pathophysiological mechanisms due to current therapies that entail the off-label use of medications to treat symptoms. 2. Despite the knowledge that the microbiota in our gut plays an essential part in maintaining gut health, their exact functions in gut homeostasis are still unclear. What constitutes a healthy microbiome and further, the precise definition of gut microbial dysbiosis is lacking. More comprehensive, large-scale, and longitudinal studies utilizing multi-omics data are needed to dissect the exact contribution of gut microbial alterations in disease pathogenesis. Accordingly, we review the current treatment options, clinical insight on pathophysiology, therapeutic modalities, current challenges, and therapeutic clues for the clinical care and management of functional dyspepsia, gastroparesis, irritable bowel syndrome, functional constipation, and functional diarrhea.

## Introduction

Functional gastrointestinal disorders (FGIDs) have been described as disorders of gut-brain interactions and are not only common in clinical practice, but also in the community (Black et al., 2020b; Sperber et al., 2021a). Recently, it has been estimated that the prevalence of FGIDs worldwide is 40% based on Rome IV criteria (Sperber et al., 2021b). However, of the 22 FGIDs as per Rome IV criteria, only a few are very common. In reference to the Rome IV criteria, the most common FGIDs are functional dyspepsia (FD) and irritable bowel syndrome (IBS) (Sperber et al., 2021a). Rome IV criteria defines FD as the presence of one or more upper abdominal symptoms: epigastric pain, epigastric burning, postprandial fullness, and early satiation and symptoms should have been active in the past 3 months, with onset at least 6 months before diagnosis; however, there should be no signs of structural disease (evaluated using upper endoscopy) that could account for these symptoms (Drossman, 2016). Additionally, FD has been divided into two sub-groups: epigastric pain syndrome (EPS) and postprandial distress syndrome (PDS) (Drossman, 2016). When symptoms (epigastric pain and burning) arise with no correlation to meal timing, it is classified as EPS, whereas when symptoms (epigastric pain, early satiation and postprandial fullness) arise or are aggravated following a meal, it is classified as PDS. Notably, there is substantial overlap between EPS and PDS subgroups. Further, as per the Rome IV criteria, IBS has been defined as altered stool frequency or form associated with abdominal pain and has occurred for a minimum of 6 months (Drossman, 2016). Further subdivision of patients occurs based on the main stool form observed using the Bristol Stool Form Scale: constipation-predominant IBS (IBS-C), diarrhea-predominant IBS (IBS-D), IBS with a mixture of stool patterns (IBS-M), and IBS with the stool pattern unclassified (IBS-U) (Drossman, 2016).

Additionally, there is substantial co-occurrence of FD and IBS (Ghoshal and Singh, 2017; Black and Ford, 2020; Ghoshal, 2020; Sperber et al., 2021b). Also, some specific GI motility disorders overlap with FD and IBS; for instance, gastroparesis overlaps with FD-PDS, similarly functional constipation has significant overlap with IBS-C and functional diarrhea with IBS-D (Parkman et al., 2011; Zikos et al., 2019). Gastroparesis is characterized by upper GI symptoms, including early satiety, vomiting, bloating, nausea, upper abdominal pain, postprandial fullness, along with delayed gastric emptying of solids without any mechanical obstruction (Grover et al., 2019; Camilleri and Sanders, 2021). Functional constipation is comprised within the clinical spectrum of IBS-C, and defecatory disorders and can be caused by colonic myopathies or motor disorders, which typically correlate with low-amplitude contractions that result in colonic stasis and impaired propulsion (Sharma et al., 2021). These altered colonic functions increase the reabsorption of water and hardening of stool, which is typically associated with a reduction in the sensation of the need to defecate. Moreover, gut motility disorders are often mistaken as FGIDs due to the subtle symptoms and the clinical and pathophysiological features it presents with.

GI motility disorders and FGIDs are diverse in nature and are intertwined with multiple pathophysiological mechanisms, such as altered gut dysmotility, intestinal barrier dysfunction, gut immune dysfunction, visceral hypersensitivity, altered GI secretion, presence and degree of bile acid (BA) malabsorption, gut microbiota dysbiosis, and altered gut-brain axis (Ghoshal and Singh, 2014; Enck et al., 2016; Enck et al., 2017; Camilleri et al., 2018; Grover et al., 2019). In these disorders, delayed or accelerated GI transit is associated with abnormal gut muscular movements (Shin et al., 2019; Singh et al., 2021b). IBS-C and functional constipation patients often have delayed GI transit, while IBS-D and functional diarrhea have accelerated GI transit (Shin et al., 2019). The underlying mechanism behind the development of FD-EPS and IBS-D is proposed to be visceral hypersensitivity while impaired fundus accommodation and delayed gastric emptying are the underlying mechanisms for FD-PDS and gastroparesis (Enck et al., 2017; Masuy et al., 2019b). Gut microbial dysbiosis leads to the activation of the gut immune response and causes epithelial barrier dysfunction, which then induces gut dysmotility and visceral hypersensitivity (Barbara et al., 2016b; Singh et al., 2021d). These findings reinforce the idea of impaired intestinal barrier function being a core pathophysiological mechanism behind FGIDs. Further, psychological comorbidities, including anxiety, depression, and stress, are often correlated with FGIDs and likely contribute to the altered pathophysiology of gut-brain interactions (Ghoshal, 2020). Understanding cellular and molecular mechanisms of underlying pathogenesis behind the gut motility disorders and FGIDs has substantially evolved during recent years. Synchronization of the enteric, parasympathetic, and sympathetic nervous system is necessary for normal control of gut function, which relies on specific GI cell types such as enteric neurons, immune cells (resident macrophages and mast cells), interstitial cells of Cajal (ICCs), enteroendocrine cells, and smooth muscle cells (SMCs) (Sanders et al., 2012; Yoo and Mazmanian, 2017). Through established animal models and human studies, the functional defects in these cells have been evidenced in the pathogenesis of these disorders (Cipriani et al., 2016; Grover et al., 2019; Mazzone et al., 2020; Singh et al., 2021a; Wei et al., 2021a). Further, these studies energized a new treatment paradigm with pharmacological agents targeting cellular and molecular defects seen in these disorders.

**FIGURE 1:**
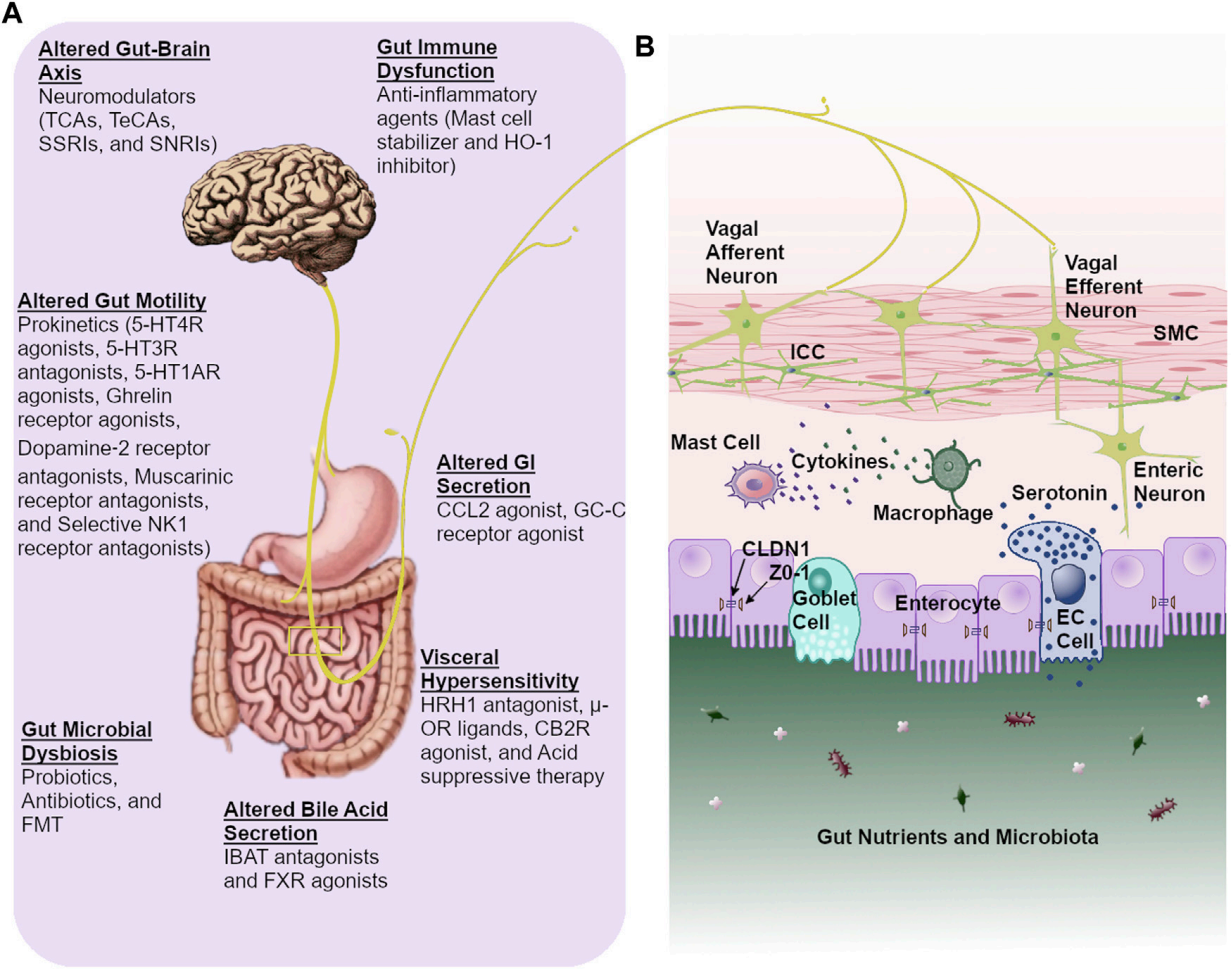
Pathophysiology-directed therapeutic approach for gastrointestinal dysmotility and functional gastrointestinal disorders. **(A)**. Currently available pharmacological agents based on pathophysiological mechanisms **(B)**. Proper gut functioning relies on a coordinated communication between intestinal epithelial cells, enteric neurons, gastrointestinal pacemaking cells, and immune cells. This allows for essential crosstalk between the gut microbiota, gut, and brain. **Abbreviations:** CCL2: chloride channel 2, CB2R: cannabinoid type 2 receptor, CLDN1: claudin 1, TCAs: tricyclic anti-depressants, TeCAs: tetracyclic anti-depressants, SSRIs: selective serotonin reuptake inhibitors, IBAT: ileal bile acid transporter, HO-1: heme oxygenase-1, 5-HT1AR: serotonin 1A receptor, NK1: neurokinin 1, FMT: fecal microbiota transplantation, FXR: farnesoid X receptor, HRH1: histamine receptor-1, µ-OR: μ-opioid receptor, GC-C: guanylate cyclase-C, ICC: interstitial cells of Cajal, EC: enterochromaffin, ZO-1: zonula occludens-1, SNRIs: serotonin noradrenaline reuptake inhibitors, SMC: smooth muscle cell.

The treatment of FGIDs and gut motility disorders has undergone a substantial paradigm shift in recent years from symptomatic treatment to subtyping of the condition and the underlying pathophysiology. Prokinetics, antispasmodics, centrally acting neuromodulators, fecal microbiota transplantation (FMT), and modified lifestyle (dietary, probiotic, and/or antibiotic interventions), are currently available treatment options for these disorders. Current proposed pharmacological agents modulating pathophysiological mechanisms include prokinetic agents (5-HT4R agonists, 5-HT3R antagonists, 5-HT1AR agonists, ghrelin receptor agonists, dopamine-2 receptor antagonists, and muscarinic receptor antagonists) for altered gut motility, mast cell stabilizer for gut immune dysfunction, acid suppression therapy and histamine receptor-1 (HRH1) antagonists for impaired duodenal clearance of gastric acid and visceral hypersensitivity, μ-Opioid receptor (μ-OR) ligands and cannabinoid type 2 receptor (CB2R) agonist for visceral pain, chloride channel 2 (CCL2) and guanylate cyclase-C (GC-C) receptor agonists for altered GI secretion, farnesoid X receptor (FXR) agonist and ileal bile acid transporter (IBAT) antagonist for altered BA secretion, central and visceral neuromodulators [tricyclic anti-depressant (TCA), tetracyclic anti-depressants (TeCA), serotonin noradrenaline reuptake inhibitors (SNRIs), and selective serotonin reuptake inhibitors (SSRIs)] for altered gut-brain axis (Drossman et al., 2018; Simren and Tack, 2018; Grover et al., 2019; Masuy et al., 2019b; Black et al., 2020b; Ghoshal, 2020; Camilleri, 2021; Sharma et al., 2021) (Figure 1). However, it is of utmost importance to unearth alternative treatment options that target the pathophysiological mechanisms underlying these conditions. Furthermore, the cause and effect cannot be disentangled among symptoms and underlying pathophysiological mechanisms due to the fact that current therapies entail the off-label use of medications to treat symptoms. More comprehensive, large-scale, and longitudinal studies utilizing multi-omics data are needed to elucidate the exact contributors in disease pathogenesis, particularly those that could be actionable with pharmacologic agents. However, it should be noted that the general lack of biomarkers, both in diagnosing FGIDs as well as for use as predictors of the patient’s response to specific treatment strategies, will likely cause further challenges for the use of “multi-omics” based longitudinal studies. Owing to the fact that a majority of current treatment options are based on symptomology, pathophysiology-based treatment might serve as a more beneficial foundation for future treatments of motility disorders and FGIDs. Accordingly, this review aims to deliberate the clinical insight on pathophysiology-directed therapeutic modalities, current challenges, and therapeutic clues, while emphasizing current gaps in knowledge as well as future directions for enhanced clinical care of motility disorders and FGIDs. In this review, we discuss FD, gastroparesis, IBS, functional constipation, and functional diarrhea. The rationale for the inclusion of only a few of these disorders is based on the recent findings such as; i. FD and IBS are the most common among all FGIDs (Sperber et al., 2021a), ii. Several reports noted FD and IBS have substantial overlap of clinical symptoms and pathophysiological mechanisms (Black and Ford, 2020), iii. FD patients (particularly FD-PDS) often overlap (90%) with gastroparesis and further, gastroparesis has clinical overlap with functional constipation (66%) (Parkman et al., 2011; Zikos et al., 2019; Ghoshal et al., 2021), iv. A recent study showed that FD and gastroparesis’s clinical features and pathological mechanisms are very similar, and the question arose if they should even be categorized as two separate conditions (Pasricha et al., 2021), and, v. Further, the management of IBS-C and functional constipation is the same. Similarly, the management of IBS-D and functional diarrhea are the same; therefore, we also covered functional constipation and functional diarrhea. For this review, literature searches were performed using PubMed from June 2021 through November 2021 to identify publications reporting on the pathophysiology, diagnostic criteria, and treatments options for FD, gastroparesis, IBS, functional constipation, and functional diarrhea. We selected 184 references for inclusion in this review due to their relevance in regard to the scope of this manuscript and based on the authors’ insight, research experience, and clinical practices in managing these disorders.

## Pathophysiological Mechanisms of Motility Disorders and FGIDs

Gut motility disorders and FGIDs are extremely diverse conditions. Therefore, understanding the molecular and cellular mechanisms underlying the pathology of these conditions is essential for the more effective treatment of these disorders. Multiple pathophysiological mechanisms are involved in the development of these disorders.

### Altered Gut Motility

Proper propulsion of food through the gut relies on peristaltic movements (Sanders et al., 2012). ICCs, enterochromaffin (EC) cells, the enteric nervous system (ENS), and GI smooth muscle cells are some of the most important factors regulating peristalsis of both longitudinal and circular smooth muscle (Grover et al., 2011; Mazzone et al., 2020; Spencer and Hu, 2020; Jin et al., 2021; Singh et al., 2021a; Singh et al., 2021b; Singh et al., 2021c; Wei et al., 2021a; Zheng et al., 2021). Further, functional defects of particular GI cells, for instance, ICCs, SMCs, EC cells, enteric neurons, and immune cells can hamper gut peristalsis (Figure 1). Gut dysmotility caused by impaired peristalsis is a key pathophysiological mechanism of these disorders (Shin et al., 2019; Spencer and Hu, 2020).

### Gut Microbial Dysbiosis

Gut microbial dysbiosis is highly overrepresented in FGIDs, particularly in IBS and FD. (Barbara et al., 2016b; Wei et al., 2021b; Singh et al., 2021d). Moreover, gut immune dysfunction, altered gut-brain axis, visceral hypersensitivity, impaired gut epithelial barrier function, altered gut motility, along with other pathophysiological mechanisms, have been demonstrated in gut microbial dysbiosis (Backhed et al., 2005; Barbara et al., 2016b; Cani, 2017; Shin et al., 2019; El-Salhy et al., 2021). Increased understanding of host-microbe interactions has shed light on the key pathophysiological role that microbes play in developing FGIDs (Schroeder and Backhed, 2016). Activation of the mucosal immune response via disruption of the gut microbial composition leads to gut barrier dysfunction, also known as the leaky gut. The development of a leaky gut also causes gut dysmotility and visceral hypersensitivity, which are pathophysiological characteristics of FGIDs (Barbara et al., 2016b; Singh et al., 2021d). While many studies have highlighted the possibility that altered gut microbial composition may trigger the development of FGIDs, it is not currently clear if this connection is more than merely a correlation (Malinen et al., 2005; Shukla et al., 2015; Ghoshal et al., 2018; Mars et al., 2020). It should be noted that disease progression can also be affected by the host diet, immune response, and host environment. Additionally, the interactions between the host and the gut microbiota or microbial-produced metabolites modulate the gut-brain physiology.

Metabolites generated by the gut microbiota continually signal to the hosts’ organs and regulate pathophysiological mechanisms during both health and disease (Sekirov et al., 2010; Schroeder and Backhed, 2016). Short-chain fatty acids (SCFAs) are key fermentation products produced by the microbiota in our gut. IBS-C patients have decreased SCFAs (propionate and butyrate), and patients with IBS-D have increased butyrate levels compared to healthy controls (HCs), emphasizing the significant role of SCFAs in regulating gut motility (Sun et al., 2019). Further, microbiota-produced SCFAs interact with EC cells and enhance the expression of tryptophan hydroxylase 1 (Tph1), which finally increases the production of serotonin in the gut of both mice and humans (Reigstad et al., 2015). Moreover, tryptamine (a monoamine, like serotonin, that is derived from tryptophan) has been shown to improve gut motility acting through 5-HT4 receptors located on colonic epithelial cells in mice (Williams et al., 2014; Bhattarai et al., 2018). Moreover, microbial dysbiosis-induced BA pool alternations might be a key pathological mechanism of FGIDs. Gut dysmotility and visceral pain are associated with elevated BA levels in IBS-D patients (Mars et al., 2020). Taken together, host physiology is greatly impacted by microbe-derived metabolites and may be targets of future therapeutic options for patients with FGIDs.

### Gut Immune Dysfunction

A subset of FGID patients have been shown to have dysfunction of their gut immune response (Muller et al., 2014; Grover et al., 2018; Black et al., 2020b; Gottfried-Blackmore et al., 2021; Ji et al., 2021). For example, an increase in the amount of immune cells in the gut (such as mast cells, T cells, macrophages, and eosinophils) of FGID patients has been reported in multiple studies (Barbara et al., 2011; Matricon et al., 2012; Barbara et al., 2016b; Singh et al., 2017). Activated mast cells release cytokines, histamines, prostaglandins, and tryptase, which are associated with intestinal barrier dysfunction and altered nociceptive pathways in FGIDs (Barbara et al., 2004; Barbara et al., 2007; Dothel et al., 2015; Aguilera-Lizarraga et al., 2021). Intestinal barrier dysfunction leads to the permeation of pathogens and food antigens, which causes a heightened immune response in the gut and greatly impacts the severity of symptoms experienced by patients with FGIDs (Camilleri et al., 2012; Wouters et al., 2016b).

### Intestinal Barrier Dysfunction

The gut luminal-mucosal interface contains food particles along with many other molecules that can induce immunogenic responses creating a constant challenge for the gut immune system (Ghoshal, 2020). As a core pathophysiological mechanism, dysfunction of the intestinal barrier activates the gut immune response, which can hamper gut function and increase the symptom severity in patients with FGIDs (Barbara et al., 2016b; Wouters et al., 2016b). The gut epithelium is an astounding barrier that allows for the selective absorption of essential nutrients, water, and electrolytes, while preventing harmful toxins, metabolites, and pathogens from penetrating the gut epithelium (Camilleri, 2019). Epithelial tight junction proteins help to maintain this selective barrier to prevent harmful molecules from penetrating the epithelium while allowing for the passage of essential nutrients (Camilleri, 2019; Motta et al., 2021). Decreased expression of these tight junction proteins, caused by genetic, pathogenic, or other factors, leads to intestinal barrier dysfunction (Bischoff et al., 2014; Desai et al., 2016; Martinez et al., 2017; Aguilera-Lizarraga et al., 2021). Several studies have shown the decreased expression of zonula occludens-1, occludin, and adhesion proteins, as well as decreased transepithelial resistance in duodenal biopsies from FGIDs patients compared to HCs, suggesting impaired intestinal barrier function in a subset of these disorders (Bertiaux-Vandaele et al., 2011; Turcotte et al., 2013; Fritscher-Ravens et al., 2014; Lee et al., 2020). Thus, healthy gut barrier integrity is critical for host defense, pathogen colonization resistance, and, more importantly, gut homeostasis.

### Visceral Hypersensitivity

Gut bacteria and their derived molecules, along with food particles are recognized and transduced through interaction with receptors on neuroimmune cells and enteroendocrine cells (Sternini et al., 2008). Visceral sensitivity is affected by the gut immune response in a manner relative to the proximity of neurons to immune cells as well as the propagation level of inflammatory reactions (Barbara et al., 2004; Aguilera-Lizarraga et al., 2021). More importantly, visceral hypersensitivity is a key pathophysiological mechanism leading to the development of FGIDs (Singh et al., 2016; Black et al., 2020b; Grover et al., 2021). Visceral hypersensitivity can be explained by an increased perception of gut mechano-chemical stimulation, which typically manifests in an aggravated feeling of pain and burning (Farzaei et al., 2016; Bellono et al., 2017). Activation of transient receptor potential vanilloid subtype 1 (TRPV1) is triggered by nerve growth factor (NGF), thermal stimulus, capsaicin, prostaglandins, acidic pH, and inflammatory mediators, which further release neuropeptides that aid in visceral pain sensation. (Farzaei et al., 2016). Furthermore, upregulation of TRPV1 has been associated with abdominal pain in IBS patients in a plethora of studies (Akbar et al., 2008; Grover et al., 2021).

### Altered Gut-Brain Interactions

Gut function is heavily influenced by the coordinated communication between the gut and brain (Quigley, 2017). This bi-directional interaction is essential for normal gut motility, visceral sensation, intestinal barrier integrity, gastric secretions, and immune response (Rhee et al., 2009). Just as the brain has a fundamental role in the maintenance of normal gut functions, the gut also has a vital role in modulating brain function. The gut-brain axis also aids in indirect signaling between the host and gut microbiota; for instance, gut microbial-induced epithelial barrier dysfunction alters this bi-directional interaction (Carabotti et al., 2015). The emotional motor system enables the perception of gut stimuli and modulates several gut functions (Needham et al., 2020). Consequently, alterations to the gut microbiota may regulate neurotransmitter synthesis or consumption, leading to emotional state and behavior alterations (Van Oudenhove et al., 2016). Many patients with FGIDs also experience psychological conditions such as stress, anxiety, and depression, indicating these conditions play a significant part in the development of FGIDs (Drossman et al., 2018). In addition, patients with FGIDs have been shown to have both abnormal structure and functional networks in parts of the brain that process information such as the visceral motor system and vagovagal reflux, as evidenced by functional brain MRI studies (Enck et al., 2017; Guleria et al., 2017; Ford et al., 2020). Taken together, it is clear that impaired brain function can lead to altered gut physiology, such as gut dysmotility and heightened visceral sensitivity, underpinning the symptoms of FGIDs. Further, dysregulated gut homeostasis can also lead to physiological changes in the brain, significantly hampering psychological health.

## Pathophysiology-Directed Therapeutic Approach for Motility Disorders and FGIDs

New therapeutics that focus on treating the underlying pathophysiological mechanisms contributing to the development of motility disorders and FGIDs are necessary to treat these patients better. FGIDs have substantial clinical overlap with specific gut motility disorders (Black and Ford, 2020; Ghoshal, 2020). Further, a multinational study conducted in 26 countries demonstrated substantial negative ramifications on the quality of life and increased psychological comorbidity for the patients who experienced multiple FGIDs (Sperber et al., 2021b). Common pathophysiological mechanisms between different FGIDs lead to the significant overlap of these conditions and is an excellent area for therapeutic development, as it would help to treat all associated underlying conditions instead of simply treating the symptoms of the conditions. Additionally, given the burden of FGIDs on the health care system, treatment options focused on the underlying pathophysiological mechanisms of FGIDs might significantly lessen the cost of patient care. In Table 1, we have summarized the mode of action and clinical outcome of current pharmacological agents based on pathophysiological mechanisms of motility disorders and FGIDs.

**TABLE 1:** Pathophysiology based pharmacological agents modulating peripheral and central factors for gastrointestinal dysmotility and functional gastrointestinal disorders.

Drug class	Pathophysiological mechanism	Mechanism of action	Clinical outcome	References
*Pharmacological agents modulating peripheral factors*				
5-HT4R agonists	Altered gut motility	5-HT4R agonists target 5-HT4Rs on interneurons and excitatory motor neurons, enhancing the release of acetylcholine, which further promotes peristalsis and secretion	Improves gut motility. Improves GI symptoms as assessed by the GCSI	Simren and Tack (2018); Ghoshal (2020); Camilleri and Atieh (2021)
5-HT3R antagonists	Altered gut motility	Patients with IBS-D have abnormal serotonergic transmission mediated through the 5-HT3Rs. Blocking 5-HTRs causes increases fluid absorption, slows gut transit, and reduces colonic contractility	Globally improves IBS symptoms, relieves abdominal pain and discomfort, and improves stool consistency and bowel movements	Simren and Tack (2018); Rokkas et al. (2021)
5-HT1AR agonists	Altered gut motility	Activation of 5-HT1AR at the level of the CNS increases gastric tone and decreases gastric sensitivity to distensionPeripheral inhibitory effect exerted by the 5-HT1AR agonist improves gastric accommodation	Enhances fundus relaxation, gastric accommodation, and improves postprandial symptoms independently from its anxiolytic effect	Tack et al. (2012)
Ghrelin receptor agonists	Altered gut motility	Stimulates ghrelin receptors that present on vagal afferents and dorsal motor nucleus of the vagus neurons innervated across the GI	Improves delayed gastric emptying in diabetic gastroparesis condition	Camilleri et al. (2020a)
Muscarinic receptor antagonists	Altered gut motility	Increases acetylcholine levels in the synaptic cleft through inhibition of acetylcholinesterase and antagonization of the presynaptic muscarinic receptors that are present on cholinergic nerve endings	Improves gut motility and is also beneficial as antispasmodics	Altan et al. (2012)
FXR agonists	Altered bile acid secretion	Inhibits hepatocyte bile acid synthesis, resulting in decreased colonic bile acid concentration	Improves stool form and symptoms of diarrhea	Walters et al. (2015); Camilleri et al. (2020b)
IBAT antagonists	Altered bile acid secretion	IBAT antagonists block the function of ASBT that is present on epithelial cells in the ileum leading to inhibition of bile acid reabsorption and subsequently increasing colonic secretion	Efficacious treatment for constipation, improving gut transit and symptoms via increasing colonic bile acids	Vijayvargiya et al. (2018)
Mast cell stabilizer	Gut immune dysfunction	The generation of hypersensitivity and gut immune dysfunction is largely influenced by mast cells	Reduces IBS symptoms by improving the visceral pain threshold in IBS patients	Klooker et al. (2010)
Histamine receptor-1 antagonists	Visceral hypersensitivity	Histamine sensitizes TRPV1+ neurons in colonic biopsies from IBS patients	Reduces visceral pain and hypersensitivity in IBS patients	Wouters et al. (2016a)
CCl2 agonists	Altered GI secretion	Increases the sodium and water secretion into the lumen by activating the CCl2 channels present on enterocytes	Improves gut motility along with the frequency and consistency of stool. Reduces abdominal pain, bloating, and straining	Drossman et al. (2009)
Guanylate cyclase-C receptor agonists	Altered GI secretion	Transmembrane GC-C receptors are located on IECs and regulate electrolyte and fluid balance in the gut and therefore help to maintain normal bowel function. Activation of GC-C receptors increases intracellular cyclic guanosine monophosphate that helps to increase colonic fluid secretion	Improves the frequency and consistency of stool and reduces straining. Reduces abdominal pain, bloating, and cramping	Rao et al. (2012)
μ-Opioid receptor ligands	Visceral hypersensitivity	Recruits *ß*-arrestin and facilitates the receptor internalization and desensitization, thereby activating μ-ORs in endosomes and inducing analgesia	Manages both severe and moderate acute pain in adults that were unable to be treated with alternative medicines (excluding opioids)	Markham, (2020)
Cannabinoid type 2 receptor agonists	Visceral hypersensitivity	CB2R agonists reduce pain as it reduces visceromotor response to colorectal distention	Has potential analgesic effects in patients with IBS	Castro et al. (2021)
*Pharmacological agents modulating central factors*				
TCAs	Altered gut-brain axis	Primarily used for anti-depressant and analgesic purposes, additionally, they can block opioid receptor activation, voltage-gated ion channels and modulate neuroimmune anti-inflammatory effects	Affects gut motility through anticholinergic and serotonergic mechanisms. Reduces visceral hypersensitivity and intestinal pain sensitivity via mediation of either peripheral nerves or the CNS	Mertz et al. (1998)
TeCAs		Boosts NA and 5-HT neurotransmission by blocking presynaptic α2-noradrenergic receptors on noradrenaline and serotonergic neurons	Orexigenic hormones are upregulated, and anorexigenic hormones are downregulated, reducing colonic hypersensitivity and improving gastric emptying	Tack et al. (2016)
SSRIs		Boosts serotonergic transmission by selective blockage of 5-HT transporter	Increases colonic motility	Tornblom and Drossman, (2015)
			Decreases symptoms of IBS scores for bloating and abdominal pain independent of centrally modulating functions	
SNRIs		Boosts NA and 5-HT neurotransmission by blocking their reuptake	Increases compliance, relaxes tone, reduces the postprandial colonic contraction, and increases sensory thresholds in response to balloon distensions	Tornblom and Drossman, (2015)
*Pharmacological agents modulating both central and peripheral factors*				
Selective NK1 receptor antagonists		Reduce the onset of emesis by affecting regions of the brain that cause vomiting and nausea through competition for NK1 receptors on vagal afferents or inhibition of major effects of substance P on key emetic pathways. Modulates the functional interplay between NK1R systems and acetylcholine, which causes stimulation of smooth muscle contractions	Improves both GCSI and nausea scores in patients with gastroparesis	Carlin et al. (2021)

Abbreviations: ASBT: apical sodium-bile acid transporter, CCl2: chloride channel 2, TCAs: tricyclic anti-depressants TeCAs: tetracyclic anti-depressants, SNRIs: serotonin noradrenaline reuptake inhibitors, CNS: central nervous system, NA: noradrenaline, NK1: neurokinin-1, 5-HTR: 5-hydroxytryptamine receptor, GCSI: gastroparesis cardinal symptom index, IBS: irritable bowel syndrome, FGF-19: fibroblast growth factor 19, IBAT: ileal bile acid transporter, TRPV1: transient receptor potential vanilloid subtype 1 DRG: dorsal root ganglion, HRH1: histamine receptor-1, IECs: intestinal epithelial cells, GC-C: Guanylate cyclase-C, μ-OR: μ-Opioid receptor, CB2R: Cannabinoid type 2 receptor, SSRIs: selective serotonin reuptake inhibitors, FXR: farnesoid X receptor.

### Pharmacological Agents Modulating Altered Gut Motility

Prokinetic medications can amplify muscular contractions in the gut to help enhance peristaltic movements of the gut; therefore, accelerating transit of intra-luminal contents (Acosta and Camilleri, 2015). Prokinetic medications can act in a generalized fashion, affecting multiple regions of the gut, or in a more specific manner, only affecting certain areas of the gut based on the location of the receptor targets (Camilleri and Atieh, 2021). 5-HT4R agonists, 5-HT3R antagonists, 5-HT1AR agonists, ghrelin receptor agonists, dopamine-2 receptor antagonists, and muscarinic receptor antagonists are all prokinetic agents that have been demonstrated to restore altered gut motility in patients with gut motility disorders and FGIDs (Simren and Tack, 2018; Grover et al., 2019; Ghoshal, 2020; Camilleri and Atieh, 2021).

### Pharmacological Agents Modulating Gut Microbial Dysbiosis

Probiotics, antibiotics, and FMT are therapeutic approaches to modulate gut microbial dysbiosis. The effectiveness of probiotic treatment for symptom improvement for FGIDs has been well documented; however, there is a lack of consistency between the current studies. Several studies have indicated improved symptom severity in patients with IBS treated with specific probiotic strains, including *Bifidobacterium lactis* DN-173 and *Bifidobacterium animalis* DN-173010154 (Aragon et al., 2010). One study observed that *Lactobacillus gasseri* OLL2716 was capable of shifting the gut microbiota community in the stomach of FD patients to comparable levels as HCs (Koga et al., 2019). Another study showed restoration of altered gut transit with symptoms improvement in IBS-C patients following probiotic treatment composed of *Bifidobacterium lactis* (Aragon et al., 2010).

Antibiotic use has also been demonstrated to improve symptom severity in patients with microbial dysbiosis-associated gut motility disorders and FGIDs (Pimentel et al., 2006; Ghoshal et al., 2011b; Ghoshal et al., 2018). For example, interventional studies reported that IBS-C and functional constipation patients have decreased breath CH_4_ following treatment with rifaximin alone or a combination of rifaximin and neomycin, which improved the constipation phenotype (Pimentel et al., 2006; Ghoshal et al., 2018). Further, FD patients treated with rifaximin demonstrated improved dyspeptic symptoms, including belching and abdominal bloating/fullness in randomized controlled trials (Iovino et al., 2014). Moreover, meta-analysis and systematic reviews have demonstrated the efficacy of rifaximin and other antibiotics in treating small intestinal bacterial overgrowth (SIBO) (Gatta and Scarpignato, 2017).

In patients with FD, there is frequently the presence of *Helicobacter pylori* (*H. pylori)* infection (40–70%) (Ghoshal et al., 2011a; Naz et al., 2013; Kim et al., 2017; Rahman et al., 2021). The exact role of *H. pylori* infection in the development of FD symptoms remains controversial and it is not clear if it is an association and/or causation (Masuy et al., 2019b). Randomized controlled trials reported substantial symptom improvement after *H. pylori* eradication therapy (McColl et al., 1998; Bruley Des Varannes et al., 2001; Malfertheiner et al., 2003; Mazzoleni et al., 2011; Kim et al., 2013; Du et al., 2016). In contrast, several studies failed to confirm convincing results for the superiority of *H. pylori* eradication therapy over placebo groups to improve FD symptoms (Talley et al., 1999a; Talley et al., 1999b; Miwa et al., 2000; Veldhuyzen van Zanten et al., 2003; Zhao et al., 2013). However, owing to the improvement of symptoms in a subset of patients with FD, eradication of *H. pylori* is recommended as a first-line therapy in *H. pylori* associated-FD patients (Camilleri and Stanghellini, 2013; Suzuki and Moayyedi, 2013; Talley and Ford, 2015; Enck et al., 2017; Suzuki, 2017).

Along with probiotics and antibiotics, FMT has been shown to restore a balanced microbiota and is a potential treatment option for gut microbial dysbiosis (Khoruts and Sadowsky, 2016). However, it is still unclear whether FMT treatment for patients with FGIDs actually helps them or merely causes a placebo effect (Pulipati et al., 2020; Shanahan et al., 2021). Further, IBS patients’ symptoms were not significantly improved following FMT treatment when compared to the placebo group in a recent meta-analysis (Ianiro et al., 2019). In contrast, IBS patients’ symptom severity was significantly improved following treatment with FMT when compared to placebo (El-Salhy et al., 2020). Therefore, larger, more meticulous, and multicentric studies are warranted to accurately assess the benefit of FMT.

### Pharmacological Agents Modulating Gut Immune Dysfunction

Increased mast cell number, pro-inflammatory M1 macrophages, and lymphocytes are characteristics of gut immune dysfunction and might be involved in the pathophysiology of gut motility disorders and FGIDs (Matricon et al., 2012). Additionally, ketotifen [A clinical trial (registration number NTR39, ISRCTN22504486) in the Netherlands], a mast cell stabilizer, was shown to increase the discomfort thresholds to rectal distension, improving abdominal pain in a subset of IBS patients (Klooker et al., 2010). However, mesalazine [ClinicalTrials.gov: NCT00626288 (phase 3)], an anti-inflammatory drug, failed to show any benefit in controlled trials on IBS (Barbara et al., 2016a). In a gastroparesis animal model, one study demonstrated that oxidative stress and damage of the pacemaker cells or enteric nerves was caused by depletion of resident M2 anti-inflammatory macrophages that express heme oxygenase-1 (HO-1) (Choi et al., 2010; Bharucha et al., 2016). However, gastric emptying was not significantly improved in a randomized-controlled trial following treatment with hemin, a HO-1-inhibitor in humans (Bharucha et al., 2016). Furthermore, patients with FD showed reduced number of mast cells and duodenal eosinophils, as well as reduced intestinal permeability and symptom severity when treated with proton pump inhibitors (Wauters et al., 2021). Further, the reduction of eosinophils was associated with clinical efficacy in these patients.

### Pharmacological Agents Modulating Visceral Hypersensitivity

A murine model of visceral hypersensitivity, along with a subset of IBS patients, demonstrated HRH1 activation, which leads to increased submucosal neuronal responses to the TRPV1-agonist, capsaicin (Wouters et al., 2016a). Ebastine [ClinicalTrials.gov: NCT01908465 (phase 4)], the HRH1-antagonist, was found to reduce abdominal pain in a state-of-the-art study on IBS patients (Wouters et al., 2016a). Visceral analgesics, such as biased μ-OR ligands and CB2R agonists have proven to improve symptoms in patients with IBS (Camilleri, 2021). G protein-mediated pathways and beta-arrestin recruitment are activated by μ-OR-agonists that induce analgesia while mediating receptor internalization and desensitization, triggering the activation of μ-ORs in endosomes, and inhibiting gut motility (Raehal et al., 2011). The μ-OR-agonist, oliceridine, has been shown to help manage moderate or severe acute pain in patients who have not seen symptom improvement while taking other treatments (excluding opioids) (Markham, 2020). Furthermore, an animal model of visceral hypersensitivity had reduced visceromotor response to colorectal distension when treated with olorinab [ClinicalTrials.gov: NCT04655599 (phase 1)], a CB2R agonist (Castro et al., 2021). More importantly, olorinab may have exceptional efficacy in IBS patients, likely due to its potential analgesic effects; however, to confirm these results, more robust studies are needed. Neuromodulators are also used for the treatment of visceral hypersensitivity in patients with FGIDs (Drossman et al., 2018; Simren and Tack, 2018). Studies have demonstrated that TCAs, particularly amitriptyline, reduce visceral hypersensitivity in patients with IBS (Poitras et al., 2002; Morgan et al., 2005; Thoua et al., 2009). Dysregulated gut-brain interaction is a major pathophysiological mechanism in patients with FGIDs. This further reinforces that central neuron degeneration might be involved in the development of abdominal pain (Tornblom and Drossman, 2015; Drossman et al., 2018).

### Pharmacological Agents Modulating Altered GI Secretion

Modulation of altered GI secretion in chronic constipation and IBS-C patients via pharmacological agents for GC-C receptors and chloride channels on intestinal epithelium cells has been well studied (Pannemans and Tack, 2018). The maintenance of bowel function through fluid and electrolyte regulation is modulated by the gut GC-C receptors expressed by intestinal epithelial cells. The activation of GC-C receptors leads to the development of an ion gradient between the gut lumen and intestinal epithelium, which stimulates water movement in the lumen through the concurrent inhibition of sodium/hydrogen exchanger isoform three channels and activation of cystic fibrosis transmembrane conductance regulator (CFTR) channels (Brancale et al., 2017). Treatment with Linaclotide (approved in most parts of the world for treating IBS-C and chronic constipation), a GC-C receptor agonist, accelerated colonic transit and softened stool in patients with IBS-C (Chey et al., 2012; Rao et al., 2012; Black et al., 2018; Islam et al., 2018).

Fluid secretion and gut motility are regulated by intestinal epithelial cells’ chloride channels (Jiang et al., 2015). Chloride ions are released into the lumen of the intestine via activation of type-2 chloride channels. This results in an ion gradient, which promotes the release of water and sodium into the gut lumen and therefore increases stool volume and accelerates gut motility. The locally acting selective type-2 chloride channel agonist Lubiprostone [United States Food and Drug Administration (U.S. FDA) approved medicine for treating patients with IBS-C and chronic constipation and is also approved for the treatment of IBS-C and chronic constipation in many other countries)] has been shown to improve the frequency and consistency of stool, which leads to reduced constipation, bloating, and straining in patients with IBS-C and functional constipation (Johanson et al., 2008; Drossman et al., 2009; Black et al., 2018).

### Pharmacological Agents Modulating Altered Bile Acid Secretion

IBAT inhibitor and FXR agonists have been proposed to modulate altered BA secretion in chronic constipation, IBS-C, and IBS-D patients (Vijayvargiya et al., 2018; Vijayvargiya and Camilleri, 2019; Khanna and Camilleri, 2021). Elobixibat [ClinicalTrials.gov: NCT01007123 (phase 2)] is an IBAT inhibitor that hinders the reabsorption of BA in the gut (Khanna and Camilleri, 2021). This results in increased concentrations of BA in the colon, promotes fluid secretion, and rescues colonic dysmotility. Treatment with Elobixibat leads to improved symptoms associated with constipation, including straining, constipation severity, stool consistency, stool frequency, and abdominal bloating in patients with IBS-C and functional constipation (Khanna and Camilleri, 2021). In contrast, since FXR agonists, obeticholic acid (US. FDA approved medicine) and tropifexor [ClinicalTrails.gov: NCT02713243 (phase 2)], inhibit hepatocyte BA synthesis, that further leads to a reduction in BA concentration in the colon’s lumen, which rescues diarrhea symptoms (Walters et al., 2015; Camilleri et al., 2020b).

### Pharmacological Agents Modulating an Altered Gut-Brain Axis

Neuromodulators TCA, TeCA, SNRIs, and SSRIs are frequently used as a second-line treatment options for patients with FGIDs, particularly IBS (Drossman et al., 2018). TCAs inhibit the reuptake of noradrenaline (NA) and 5-HT and have demonstrated potential for their anti-depressant and analgesic effects (Tornblom and Drossman, 2015). In contrast, TeCAs block presynaptic *α*2-noradrenergic receptors on NA and 5-HT neurons resulting in increased NA and 5-HT neurotransmission (Tornblom and Drossman, 2015). SSRIs boost the neurotransmission of 5-HT by blocking the presynaptic 5-HT transporter. However, the effect of these drugs is more beneficial in the treatment of psychological disorders than chronic pain syndromes. Finally, SNRIs block the reuptake of both the 5-HT and NA, boosting their neurotransmission and modulating pain sensation (Tornblom and Drossman, 2015).

These findings suggest that pathophysiology-directed therapeutic modalities could provide precise clinical outcomes in FGIDs as there is significant clinical overlap among FGIDs.

## Current Treatment Options for Motility Disorders and FGIDs

The best treatment approach for motility disorders and FGIDs would be to fix the cellular and molecular defects linked with the pathophysiological mechanisms, which would also improve cardinal GI symptoms. However, GI symptoms in motility disorders and FGIDs may not always reflect the underlying pathophysiological mechanisms. Further, without knowing the exact pathologies, the treatment approach might be ineffective and result in higher healthcare expenditure and poor patient quality of life, suggesting that pathophysiology-directed therapeutic strategies might be a better therapeutic approach. Here, we have discussed available treatment options and pathophysiology-based therapeutic options for gastroduodenal motility disorders (FD and gastroparesis) and bowel disorders (IBS-C/functional constipation and IBS-D/functional diarrhea). Some medications discussed in this review for motility disorders and FGIDs are not FDA approved but are approved and available for clinical use in Europe, Asia, and/or Latin America.

### Candidate Drugs for Gastroduodenal Motility Disorders

In Table 2, we have summarized the candidate drugs for gastroparesis and FD.

**TABLE 2:** Currently available pharmacological agents for gastroduodenal motility disorders.

Class/Drug name		Status (clinically approved/development/availability)	Clinical outcome	Disease condition	References
Prokinetics (5-HT4R agonists, Selective 5-HT4R agonists, 5-HT1AR agonists, Ghrelin receptor agonists, Dopamine-2 receptor antagonists, Muscarinic receptor antagonists**)**	Prucalopride	Approved for chronic constipation in most parts of the world. It shows efficacy in patients with idiopathic gastroparesis	Improves GI symptoms as assessed by the GCSI. Improves solid gastric emptying	Gastroparesis, FD	Camilleri et al. (2008); Carbone et al. (2019); Grover et al. (2019)
	Felcisetrag	Phase 2 study in gastroparesis suggested clinical efficacy in idiopathic and diabetic gastroparesis	Accelerates gut transit in patients with gastroparesis. Stimulates secretion and motility and by enhancing the release of acetylcholine from interneurons and excitatory motor neurons	Gastroparesis	Chedid et al. (2021)
	Buspirone	Phase 2 study in gastroparesis demonstrated clinical efficacy in gastroparesis	Fundus relaxation and improves gastric accommodation	Gastroparesis, FD	Miwa et al. (2009); Tack et al. (2012); Tack et al. (2016)
	Mirtazapine	Phase 4 study in FD showed improvement in global dyspeptic symptoms and early satiation nausea in patients with FD.			
	Relamorelin	U.S. FDA has granted Fast Track designation for the treatment of diabetic gastroparesis	Stimulates nodose afferents and DMV neurons and accelerates gastric emptying	Gastroparesis	Camilleri et al. (2020a)
	Metoclopramide Domperidone Itopride	Metoclopramide: (U.S. FDA approved medicine), Domperidone: (not approved in United States , but can be used through an FDA IND; however, it is available in most other parts of the world with restricted usage recommendations due to concerns over QT prolongation risk), and Itopride: (available mainly in Asia and Eastern Europe) are D2-receptor antagonists that exert both prokinetic and antiemetic effects in patients with gastroparesis and FD	Improves gut motility	FD, gastroparesis	Patterson et al. (1999); Dumitrascu and Weinbeck (2000); Masuy et al. (2019b)
	Acotiamide	Approved in Japan and India for FD.Phase 2 long-term safety study in FD was completed in the United States and Europe	Cholinergic nerve endings express presynaptic muscarinic receptors, which are inhibited by acetylcholinesterase, leading to increased acetylcholine levels in the synaptic cleft	FD	Altan et al. (2012)
Acid suppressive therapy	Pantoprazole	Approved worldwide for acid-related disorders (peptic ulcer, reflux esophagitis, reflux disease), *H. pylori* eradication and Zollinger-Ellison syndrome). In a phase 4 study, pantoprazole showed clinical efficacy in FD	Improves intestinal permeability by reducing mast cells and eosinophils in duodenum biopsy from FD patients	FD	Masuy et al. (2019b); Wauters et al. (2021)

Abbreviations: 5-HT1AR: 5-hydroxytryptamine receptor 1A, GCIS: gastroparesis cardinal symptom index, FD: functional dyspepsia, IBS-C: constipation-predominant IBS, DMV: dorsal motor nucleus of the vagus.

## Prokinetic Agents

Based on previous clinical trials and meta-analyses, prokinetics have been recommended to treat gastroparesis, FD, IBS-C, and chronic constipation (Simren and Tack, 2018; Carbone et al., 2019; Grover et al., 2019; Camilleri and Atieh, 2021; Sharma et al., 2021). The pharmacological classes of prokinetics include serotonin 5-HT4R agonists, 5-HT1AR agonists, ghrelin receptor agonists, dopamine-2 receptor antagonists, and muscarinic receptor antagonists.

### 5-HT4R Agonists

Prucalopride [ClinicalTrials.gov: NCT02031081 (phase 2), NCT02510976 (phase 4)], a 5-HT4R agonist, exerts both gastro- and enterokinetic effects, which improves GI symptoms when assessed by the gastroparesis cardinal symptom index (GCSI) (Carbone et al., 2019). Felcisetrag [ClinicalTrials.gov: NCT03281577 (phase 2)] and Velusetrag [ClinicalTrials.gov: NCT01718938 (phase 2)] are additional selective 5-HT4R agonists that have gut prokinetic effects (Chedid et al., 2021; Kuo et al., 2021). They were shown to induce symptom relief and accelerate GI transit in idiopathic or diabetic gastroparesis patients (Chedid et al., 2021; Kuo et al., 2021). Defects in gut motility, including impaired fundus accommodation and delayed gastric emptying, have been shown in a subset of patients with FD (Asano et al., 2017; Enck et al., 2017). Further, treatment with prokinetic agents typically restores impaired gastric-duodenal motility in patients with FD-PDS (Masuy et al., 2019b).

### 5-HT1AR Agonists

Buspirone [ClinicalTrials.gov: NCT03587142 (phase 2)], a 5-HT1AR agonist, improves gastric accommodation and postprandial symptoms in patients with FD (Tack et al., 2012). Another 5-HT1AR agonist, Tandospirone, has shown significant resolution in FD symptoms compared to the placebo-treated group in a multicenter study (Miwa et al., 2009). Mirtazapine [ClinicalTrials.gov: NCT01240096 (phase 4)]), a TeCA with 5-HT1AR agonist activity on central and peripheral 5-HT1AR, leads to gastric fundus relaxation with improved nutrient volume tolerance, global dyspeptic symptoms, and early satiation nausea in patients with FD (Tack et al., 2016). Further, recent reports noted that mirtazapine improves nausea, vomiting, and loss of appetite in patients with gastroparesis (Malamood et al., 2017; Marella et al., 2019).

### Ghrelin Receptor Agonists

Relamorelin (US. FDA approved medicine), a ghrelin receptor agonist, stimulates nodose afferents and dorsal motor nucleus of the vagus neurons, while accelerating gastric emptying and improving pain, nausea, bloating, and fullness in diabetic gastroparesis patients (Chedid and Camilleri, 2017; Camilleri et al., 2020a).

### Dopamine-2 Receptor Antagonists

Metoclopramide (US. FDA approved medicine), domperidone (not approved in United States , but can be used through an FDA IND, due to concerns over its cardiac side effects. In Europe, domperidone has long been available over the counter; however, based on recent concerns over risk for prolongation of the QT-interval and increased risk of ventricular arrhythmia, it has only limited availability in Europe on a prescription basis, and only short-term use is recommended), and itopride [ClinicalTrials.gov: NCT00110968 (phase 3)], available mainly in Asia and to some extent in Eastern Europe are D2-receptor antagonists that exert both prokinetic and antiemetic effects in patients with gastroparesis and FD (Patterson et al., 1999; Dumitrascu and Weinbeck, 2000; Masuy et al., 2019b). Treatment with metoclopramide may result in adverse reactions, including a very serious condition called tardive dyskinesia. Additionally, there is an FDA black box warning for long-term use of metoclopramide (Rao and Camilleri, 2010).

### Muscarinic Receptor Antagonists

Acotiamide [ClinicalTrials.gov: NCT03402984 (phase 2)], a muscarinic receptor antagonist (acetylcholinesterase inhibitor), antagonizes the presynaptic muscarinic receptors and has a prokinetic effect throughout the gut (Altan et al., 2012; Masuy et al., 2019a) and has been developed and approved in Japan and India for the treatment of FD. Studies have shown its efficacy in improving impaired gastric accommodation in patients with FD (Altan et al., 2012; Matsueda et al., 2012).

## Selective NK1 Receptor Antagonists

There is extraordinary potential for neurokinin-1 (NK1) or tachykinin receptor antagonists since they are effective both as antiemetics and gastrokinetics (Carlin et al., 2021). As antiemetic agents, they reduce the onset of emesis by affecting regions of the brain that cause vomiting and nausea through competition for NK1 receptors on vagal afferents or inhibition of significant effects of substance P on key emetic pathways. As gastrokinetics, they stimulate smooth muscle contractions in the stomach (Jacob et al., 2017). Tradipitant [ClinicalTrials.gov: NCT04028492 (phase 3)], a NK1 receptor antagonist, has been tested in patients with gastroparesis, and it showed improvement in GCSI, particularly nausea and vomiting scores (Jacob et al., 2017; Carlin et al., 2021).

## Acid Suppressive Therapy

Patients with FD have duodenal hypersensitivity to gastric acid as well as impaired duodenal clearance of gastric acid, which highlights acid suppression therapy as a possible treatment for FD (Moayyedi et al., 2004). Acid suppression therapy is approved worldwide for acid-related disorders (peptic ulcer, reflux esophagitis, reflux disease, *H. pylori* eradication and Zollinger-Ellison syndrome). The first-line therapy for FD patients is acid suppression with proton pump inhibitors (PPIs), for instance lansoprazole, rabeprazole, pantoprazole, and omeprazole, although this may be the most commonly effective for the groups that have both EPS-FD and FD-GERD (Masuy et al., 2019b). Furthermore, a proof-of-concept study showed that pantoprazole [ClinicalTrials.gov: NCT03545243 (phase 4)] reduced duodenal eosinophils, mast cells, and intestinal permeability, which were correlated with a better clinical outcome in FD patients (Wauters et al., 2021).

### Candidate Drugs for Bowel Disorders

In Table 3, we have summarized the candidate drugs for IBS, functional constipation, and functional diarrhea.

**TABLE 3:** Currently available pharmacological agents for bowel disorders.

Class/Drug name		Status (clinically approved/ development/availability)	Clinical outcome	Disease condition	References
Prokinetics (5-HT4R agonists, 5-HT3R antagonists)	Prucalopride, Tegaserod	Prucalopride is approved for chronic constipation in most parts of the world.The U.S. FDA approved reintroduction of tegaserod in 2019 for female patients (younger than 65 years old) with IBS-C	Improves GI symptoms as assessed by the GCSI. Improves solid gastric emptying	IBS-C/ functional constipation	Camilleri et al. (2008)
	Alosetron, Ramosetron, Ondasetron	Alosetron: FDA approved to treat only female patients with IBS-D. Ramosetron: phase 4 clinical trial showed clinical efficacy in male patients with IBS-D. Ondasetron: phase 3 clinical trial demonstrated clinical efficacy in patients with IBS-D, irrespective of gender	Improves stool consistency and bowel movements	IBS-D/functional diarrhea	Rokkas et al. (2021)
Antibiotics	Rifaximin	Approved in most parts of the world for the treatment of IBS. Rifaximin showed clinical efficacy in FD patients	Shifts the microbial community composition. Improves constipation, SIBO, and dyspeptics symptoms	IBS, FD	Pimentel et al. (2011); Ghoshal et al. (2018)
Probiotics	*Bifidobacterium lactis* DN-173, *Lactobacillus gasseri, Lactobacillus gasseri* OLL2716, *Bifidobacterium bifidum* YIT10347,	Not approved. Emerging research on probiotics demonstrated symptom improvement in patients with IBS and FD. However, probiotic intervention is an active area of research, and clinical outcomes of probiotic strains in clinical trials for IBS and FD are eagerly awaited	Modulates gut microbiota profile. Improves symptoms and gut transit	IBS, FD	Aragon et al. (2010); Charbonneau et al. (2013); Connell et al. (2018)
Bile acid sequestrants (FXR agonists)	Obeticholic acid, Tropifexor	Obeticholic acid: U.S. FDA approved for treating primary biliary cholangitis Tropifexor: showed clinical efficacy in phase 2 clinical trials composed of patients with primary bile acid diarrhea	Inhibits hepatic bile acid synthesis and improves the stool index of patients with bile acid-associated diarrhea	IBS-D/functional diarrhea	Walters et al. (2015); Camilleri et al. (2020b)
Bile acid transporter inhibitor (IBAT antagonists)	Elobixibat	Approved in Japan for treating chronic constipation. Elobixibat demonstrated clinical efficacy in phase 2 clinical trials composed of patients with chronic constipation	Efficacious treatment for constipation, improves gut transit and symptoms via increasing colonic bile acids	IBS-C/ functional constipation	Vijayvargiya et al. (2018)
Anti-inflammatory agents (Mast cell stabilizer, Histamine receptor-1 antagonist)	Mesalazine	Phase 3 clinical trial failed to show any benefit in patients with IBS	Sustains therapy response and benefits for a subgroup of patients with IBS in maintaining gut immune homeostasis	IBS	Barbara et al. (2016a)
	Ketotifen	A clinical trial (registration number NTR39, ISRCTN22504486) in the Netherlands showed increased discomfort thresholds to rectal distension, resulting in improved abdominal pain in a subset of IBS patients	Reduces symptoms by improving visceral pain threshold levels in IBS patients	IBS	Klooker et al. (2010)
	Ebastine	Phase 4 clinical trials showed clinical efficacy in patients with IBS	Reduces abdominal pain and visceral hypersensitivity in patients with IBS	IBS	Wouters et al. (2016a)
Neuromodulators (TCAs, TeCAs, SSRIs, SNRIs)	Amitriptyline	Phase 2 clinical trials showed improvement of GI symptoms and sleep quality in patients with FD. It reduces visceral hypersensitivity in patients with IBS	Affects gastrointestinal motility through anticholinergic and serotonergic mechanisms. TCAs reduce visceral hypersensitivity. Anti-depressant therapy may lead to neurogenesis	FD, IBS	Mertz et al. (1998)
	Duloxetine	Phase 4 clinical trials showed clinical efficacy in patients with IBS	Improved GI symptom severity via indirectly treating depressive symptoms	IBS	Drossman et al. (2018)
					Tornblom and Drossman, (2018)
Intestinal Secretagogues (CCl2 agonists, Guanylate cyclase-C receptor agonists)	Lubiprostone	U.S. FDA approved medicine for treating patients with IBS-C and chronic constipation and is also approved for the treatment of IBS-C and chronic constipation in many other countries	Increases fecal water content by promoting fluid secretion into the lumen	IBS-C/ functional constipation	Drossman et al. (2009)
	Linaclotide	Approved in most parts of the world for the treatment of IBS-C and chronic constipation	Increases water secretion via targeting cGMP leading to the secretion of bicarbonate and chloride into the gut	IBS-C/ functional constipation	Chey et al. (2012)
Visceral Analgesics (Biased μ-Opioid receptor ligands, CB2R agonists)	Oliceridine	U.S. FDA approved medicine for managing moderate to severe acute pain in adults. It has comparable analgesic effects to morphine, although human studies are necessary to test the efficacy for visceral pain management in patients with IBS	Manages severe and moderate acute pain in adults that were unable to be successfully treated with other medications (excluding opioids)	IBS	Markham (2020); Camilleri (2021)
	Olorinab	Phase 1 clinical trials demonstrated clinical efficacy in IBS patients, likely due to its potential analgesic effects	Potential analgesic effects in patients with IBS. More robust studies are needed to test the efficacy	IBS	Castro et al. (2021)

**Abbreviations:** 5-HT3R: 5-hydroxytryptamine receptor 3, cGMP: cyclic guanosine monophosphate, GCSI: gastroparesis cardinal symptom index, IBS-D: diarrhea-predominant IBS, CB2R: cannabinoid type 2 receptor, FGF-19: fibroblast growth factor 19, SNRIs: serotonin noradrenaline reuptake inhibitors, TCAs: tricyclic anti-depressants, CCl2: chloride channel 2, TeCAs: tetracyclic anti-depressants, IBAT: ileal bile acid transporter, SIBO: small intestinal bacterial overgrowth, SSRIs: selective serotonin reuptake inhibitors, IBS-C: constipation-predominant IBS.

## Prokinetics

### 5-HT4R Agonists

5-HT4R agonists accelerate gut transit (Simren and Tack, 2018; Ghoshal, 2020). Gut motility was substantially improved in IBS-C patients treated with Tegaserod when compared to the placebo-treated patients (Black et al., 2020a) (Johanson et al., 2004). This drug led to a slight increase in cardiovascular and cerebrovascular ischaemic events, and therefore use was discontinued. The US. FDA approved reintroduction of tegaserod in 2019 for female patients with IBS that had no history of cardiovascular disease and were younger than 65 years old (Shah et al., 2021). Further, there were no significant cardiovascular events related to tegaserod observed in patients with ≤1 cardiac risk factor in this study. Prucalopride (approved for chronic constipation in most parts of the world) is another 5-HT4R agonist that demonstrated significantly improved chronic-constipation symptoms when compared to the placebo-treated group; however, IBS-C studies lack randomized controlled trials (Camilleri et al., 2008).

### 5-HT3R Antagonists

Alosetron (FDA approved medicine), ramosetron [ClinicalTrials.gov: NCT01225237 (phase 4)], and ondansetron [ClinicalTrails.gov: NCT03555188 (phase 3)], 5-HT3R antagonists, have been demonstrated to effectively treat IBS-D patients (Simren and Tack, 2018). Among 18 randomized controlled trials 5-HT3R antagonists were the best at improving stool consistency and symptoms of IBS-D, such as abdominal pain (Rokkas et al., 2021).

## Probiotics and Antibiotics

Rifaximin (approved in most parts of the world for the treatment of IBS) was studied in randomized trials involving 1,260 patients with IBS (Pimentel et al., 2011). It was demonstrated to improve overall symptoms and bloating when compared to the placebo group. A study on FD patients also showed the efficacy of rifaximin, which was tolerated well and provided adequate relief of dyspeptic symptoms when compared to placebo treated patients (Tan et al., 2017).

Adults experiencing upper GI symptoms who ingested fermented milk with or without *Bifidobacterium bifidum* YIT10347 daily had improved abdominal discomfort and epigastric pain (Gomi et al., 2018). Another interventional study with *Lactobacillus gasseri* OLL2716 or placebo on FD patients showed symptom improvement in the treated group (Koga et al., 2019). *Bifidobacterium infantis* 35,624 has been found to normalize bowel movements and improve overall symptoms in all IBS subtypes (Charbonneau et al., 2013). *Bifidobacterium lactis* DN-173010 and VSL#3 probiotics are effective in treating flatulence, distention, and bloating (Aragon et al., 2010; Connell et al., 2018).

## Bile Acid Sequestrants

Adults with symptoms characteristic of IBS-D and functional diarrhea were shown to have an overrepresentation of increased fecal BA excretion (Vijayvargiya and Camilleri, 2019; Mars et al., 2020). Obeticholic acid stimulates fibroblast growth factor 19 and results in a reduction of colonic BA concentration, therefore rescuing diarrhea (Walters et al., 2015). Moreover, Tropifexor slowed colonic emptying and improved the stool index of patients with BA-associated diarrhea (Camilleri et al., 2020b). Finally, an additional FXR agonist, obeticholic acid got US. FDA approval for treating primary biliary cholangitis (Walters et al., 2015; Camilleri et al., 2020b; Bowlus et al., 2020).

## Bile Acid Transporter Inhibitor

Elobixibat, an IBAT inhibitor, improves colonic motility in patients with IBS-C and functional constipation (Chey et al., 2011; Vijayvargiya et al., 2018; Khanna and Camilleri, 2021).

## Anti-inflammatory Agents

The occurrence of visceral hypersensitivity in some patients with IBS and has been associated with the release of tryptase and histamine, mucosal mast cell activation (Matricon et al., 2012; Aguilera-Lizarraga et al., 2021) Ketotifen improved the visceral pain threshold and reduced abdominal pain in IBS patients with visceral hypersensitivity (Klooker et al., 2010). Another study showed that the HRH1 antagonist, ebastine, reduced visceral hypersensitivity, and further, patients also experience symptom relief when compared to the placebo group (Wouters et al., 2016a).

## Intestinal Secretagogues

Intestinal secretagogues, Lubiprostone and linaclotide (approved medicine for treating patients with IBS-C and chronic constipation and is also approved for the treatment of IBS-C and chronic constipation in many other countries) demonstrated exceptional efficacy in patients with IBS-C and functional constipation (Pannemans and Tack, 2018). Lubiprostone increases the amount of fluid secretion into the gut through bicarbonate and chloride secretion by acting on intestinal enterocytes ClC2, which leads to accelerated gut transit and improved stool consistency (Johanson et al., 2008; Drossman et al., 2009; Black et al., 2018). Linaclotide accelerates gut transit and inhibits visceral hypersensitivity by acting on the GC-C receptor on enterocytes (Chey et al., 2012; Rao et al., 2012; Black et al., 2018).

## Visceral Analgesics

The GI tract contains a plethora of opioid receptors, and drugs target these receptors to reduce the perception of pain and slow gut transit (Raehal et al., 2011). Analgesia is induced through G protein-mediated pathway activation or *ß*-arrestin activation when opioids bind to μ-opioid receptors, which depresses central nervous system functions (e.g., respiration and cognition) and delays gut motility (Raehal et al., 2011). Biased μ-opioid receptor ligands can improve gut function and analgesia by solely activating the G protein pathway (Camilleri, 2021). The biased μ-opioid receptor ligand, oliceridine, has comparable analgesic effects to morphine, although human studies are necessary to test the efficacy for visceral pain management (Singla et al., 2019; Markham, 2020). Olorinab is a CB2 agonist that alters immune function and visceral sensation in a rodent model of colitis and might modulate gut motility in patients with IBS (Castro et al., 2021). In the future, human studies are warranted to test these medications in treating visceral pain in patients with FGIDs and gut motility disorders.

## Central Neuromodulators

Central neuromodulators are increasingly used for the treatment of FGIDs (Drossman et al., 2018). One study on patients with FD showed amitriptyline [ClinicalTrilas.gov: NCT00248651 (phase 2)], a TCA, improved GI symptoms and modestly improved sleep quality (Mertz et al., 1998; Herrick et al., 2018). TCAs were more efficacious in 11 randomized trials involving 744 patients with chronic constipation and IBS-C when compared to the placebo group (Ford et al., 2014). TCAs are first-line central neuromodulators that can be used to treat IBS, particularly IBS-D. Further, poor sleep and diarrhea were improved following treatment with the TCAs (imipramine and amitriptyline) (Rahimi et al., 2009). The SSRI [Citalopram, ClinicalTrials.gov: NCT00477165 (phase 2)] and SNRI [duloxetine, ClinicalTrials.gov: NCT00401258 (phase 4); or milnacipran, Clinicaltrials. gov: NCT01471379 (phase 2)] class of medications can reduce pain in patients with IBS (Brennan et al., 2009; Tornblom and Drossman, 2018). In addition, they have fewer side effects than TCAs; however, more robust studies are warranted to elucidate the effects of these medications in patients with IBS.

## Conclusion and Future Directions

An enhanced understanding of the physiological and pathophysiological mechanisms underlying functional and motility GI disorders has ushered in the development of novel treatment approaches in the clinical care of patients. Pharmacological agents that are developed based on the cellular and molecular mechanisms underlying pathologies of these disorders provide the best avenue for future pharmaceutical development. Additionally, currently available therapies lack long-term effectiveness and safety and have poorly understood mechanisms of action for their use to treat motility disorders and FGIDs. The heterogeneous nature, the poor correlation between improved gut functions and symptoms, and the absence of a single unifying target mechanism are hurdles to developing new therapeutic options.

The collaborative work between gastroenterologists, microbiologists, neurologists, epidemiologists, and bioinformaticians may lead to thrilling discoveries in the field of FGIDs. Further, the enhanced understanding of host-gut microbial crosstalk will allow better diagnostics and treatment options for patients with these disorders. Due to substantial clinical overlap between these disorders and the sharing of symptoms and pathophysiological mechanisms between different anatomical GI regions, a combination of symptoms along with testing for the underlying cellular and molecular pathologies might help physicians stratify subsets of patients that can be more effectively treated using specific medications.

An innovative approach that uses longitudinal and multicentric studies aims to fill current knowledge gaps and characterize the patients precisely based on multi-omics data profiling from the host epigenome, transcriptome, dietary profiles, metabolome, and gut microbiome, allowing for more effective treatment of these patients. More targeted approaches will help to relieve symptoms and restore gut-brain homeostasis in patients while enhancing the stratification of therapeutic modalities for gut motility disorders and FGIDs.

## References

[B1] Acosta A. Camilleri M. (2015). Prokinetics in Gastroparesis. Gastroenterol. Clin. North. Am. 44 (1), 97–111. 10.1016/j.gtc.2014.11.008 25667026

[B2] Aguilera-Lizarraga J. Florens M. V. Viola M. F. Jain P. Decraecker L. Appeltans I. (2021). Local Immune Response to Food Antigens Drives Meal-Induced Abdominal Pain. Nature 590 (7844), 151–156. 10.1038/s41586-020-03118-2 33442055 PMC7610810

[B3] Akbar A. Yiangou Y. Facer P. Walters J. R. Anand P. Ghosh S. (2008). Increased Capsaicin Receptor TRPV1-Expressing Sensory Fibres in Irritable Bowel Syndrome and Their Correlation with Abdominal Pain. Gut 57 (7), 923–929. 10.1136/gut.2007.138982 18252749 PMC2564830

[B4] Altan E. Masaoka T. Farré R. Tack J. (2012). Acotiamide, a Novel Gastroprokinetic for the Treatment of Patients with Functional Dyspepsia: Postprandial Distress Syndrome. Expert Rev. Gastroenterol. Hepatol. 6 (5), 533–544. 10.1586/egh.12.34 23061703

[B5] Aragon G. Graham D. B. Borum M. Doman D. B. (2010). Probiotic Therapy for Irritable Bowel Syndrome. Gastroenterol. Hepatol. (N Y) 6 (1), 39–44. 20567539 PMC2886445

[B6] Asano H. Tomita T. Nakamura K. Yamasaki T. Okugawa T. Kondo T. (2017). Prevalence of Gastric Motility Disorders in Patients with Functional Dyspepsia. J. Neurogastroenterol Motil. 23 (3), 392–399. 10.5056/jnm16173 28423481 PMC5503289

[B7] Bäckhed F. Ley R. E. Sonnenburg J. L. Peterson D. A. Gordon J. I. (2005). Host-bacterial Mutualism in the Human Intestine. Science 307 (5717), 1915–1920. 10.1126/science.1104816 15790844

[B8] Barbara G. Cremon C. Annese V. Basilisco G. Bazzoli F. Bellini M. (2016a). Randomised Controlled Trial of Mesalazine in IBS. Gut 65 (1), 82–90. 10.1136/gutjnl-2014-308188 25533646 PMC4717362

[B9] Barbara G. Cremon C. Carini G. Bellacosa L. Zecchi L. De Giorgio R. (2011). The Immune System in Irritable Bowel Syndrome. J. Neurogastroenterol Motil. 17 (4), 349–359. 10.5056/jnm.2011.17.4.349 22148103 PMC3228974

[B10] Barbara G. Stanghellini V. De Giorgio R. Cremon C. Cottrell G. S. Santini D. (2004). Activated Mast Cells in Proximity to Colonic Nerves Correlate with Abdominal Pain in Irritable Bowel Syndrome. Gastroenterology 126 (3), 693–702. 10.1053/j.gastro.2003.11.055 14988823

[B11] Barbara G. Wang B. Stanghellini V. de Giorgio R. Cremon C. Di Nardo G. (2007). Mast Cell-dependent Excitation of Visceral-Nociceptive Sensory Neurons in Irritable Bowel Syndrome. Gastroenterology 132 (1), 26–37. 10.1053/j.gastro.2006.11.039 17241857

[B12] Barbara G. Feinle-Bisset C. Ghoshal U. C. Santos J. Vanner S. J. Vergnolle N. (2016b). The Intestinal Microenvironment and Functional Gastrointestinal Disorders. Gastroenterology 150, 1305–1318. 10.1053/j.gastro.2016.02.028 27144620

[B13] Bellono N. W. Bayrer J. R. Leitch D. B. Castro J. Zhang C. O'Donnell T. A. (2017). Enterochromaffin Cells Are Gut Chemosensors that Couple to Sensory Neural Pathways. Cell 170 (1), 185–e16. e116. 10.1016/j.cell.2017.05.034 28648659 PMC5839326

[B14] Bertiaux-Vandaële N. Youmba S. B. Belmonte L. Lecleire S. Antonietti M. Gourcerol G. (2011). The Expression and the Cellular Distribution of the Tight junction Proteins Are Altered in Irritable Bowel Syndrome Patients with Differences According to the Disease Subtype. Am. J. Gastroenterol. 106 (12), 2165–2173. 10.1038/ajg.2011.257 22008894

[B15] Bharucha A. E. Daley S. L. Low P. A. Gibbons S. J. Choi K. M. Camilleri M. (2016). Effects of Hemin on Heme Oxygenase-1, Gastric Emptying, and Symptoms in Diabetic Gastroparesis. Neurogastroenterol Motil. 28 (11), 1731–1740. 10.1111/nmo.12874 27283929 PMC5083191

[B16] Bhattarai Y. Williams B. B. Battaglioli E. J. Whitaker W. R. Till L. Grover M. (2018). Gut Microbiota-Produced Tryptamine Activates an Epithelial G-Protein-Coupled Receptor to Increase Colonic Secretion. Cell Host Microbe 23 (6), 775–e5. 10.1016/j.chom.2018.05.004 29902441 PMC6055526

[B17] Bischoff S. C. Barbara G. Buurman W. Ockhuizen T. Schulzke J. D. Serino M. (2014). Intestinal Permeability-Aa New Target for Disease Prevention and Therapy. BMC Gastroenterol. 14, 189. 10.1186/s12876-014-0189-7 25407511 PMC4253991

[B18] Black C. J. Burr N. E. Ford A. C. (2020a). Relative Efficacy of Tegaserod in a Systematic Review and Network Meta-Analysis of Licensed Therapies for Irritable Bowel Syndrome with Constipation. Clin. Gastroenterol. Hepatol. 18 (5), 1238–e1. e1231. 10.1016/j.cgh.2019.07.007 31302307

[B19] Black C. J. Burr N. E. Quigley E. M. M. Moayyedi P. Houghton L. A. Ford A. C. (2018). Efficacy of Secretagogues in Patients with Irritable Bowel Syndrome with Constipation: Systematic Review and Network Meta-Analysis. Gastroenterology 155 (6), 1753–1763. 10.1053/j.gastro.2018.08.021 30144426

[B20] Black C. J. Drossman D. A. Talley N. J. Ruddy J. Ford A. C. (2020b). Functional Gastrointestinal Disorders: Advances in Understanding and Management. Lancet 396 (10263), 1664–1674. 10.1016/S0140-6736(20)32115-2 33049221

[B21] Black C. J. Ford A. C. (2020). Global burden of Irritable Bowel Syndrome: Trends, Predictions and Risk Factors. Nat. Rev. Gastroenterol. Hepatol. 17 (8), 473–486. 10.1038/s41575-020-0286-8 32296140

[B22] Bowlus C. L. Pockros P. J. Kremer A. E. Parés A. Forman L. M. Drenth J. P. H. (2020). Long-Term Obeticholic Acid Therapy Improves Histological Endpoints in Patients with Primary Biliary Cholangitis. Clin. Gastroenterol. Hepatol. 18 (5), 1170–e6. e1176. 10.1016/j.cgh.2019.09.050 31606455

[B23] Brancale A. Shailubhai K. Ferla S. Ricci A. Bassetto M. Jacob G. S. (2017). Therapeutically Targeting Guanylate Cyclase-C: Computational Modeling of Plecanatide, a Uroguanylin Analog. Pharmacol. Res. Perspect. 5 (2), e00295. 10.1002/prp2.295 28357122 PMC5368960

[B24] Brennan B. P. Fogarty K. V. Roberts J. L. Reynolds K. A. Pope H. G. Jr. Hudson J. I. (2009). Duloxetine in the Treatment of Irritable Bowel Syndrome: an Open-Label Pilot Study. Hum. Psychopharmacol. 24 (5), 423–428. 10.1002/hup.1038 19548294

[B25] Bruley Des Varannes S. Fléjou J. F. Colin R. Zaïm M. Meunier A. Bidaut-Mazel C. (2001). There Are Some Benefits for Eradicating *Helicobacter pylori* in Patients with Non-ulcer Dyspepsia. Aliment. Pharmacol. Ther. 15 (8), 1177–1185. 10.1046/j.1365-2036.2001.01014.x 11472320

[B26] Camilleri M. Atieh J. (2021). New Developments in Prokinetic Therapy for Gastric Motility Disorders. Front. Pharmacol. 12, 711500. 10.3389/fphar.2021.711500 34504426 PMC8421525

[B27] Camilleri M. Chedid V. Ford A. C. Haruma K. Horowitz M. Jones K. L. (2018). Gastroparesis. Nat. Rev. Dis. Primers 4 (1), 41. 10.1038/s41572-018-0038-z 30385743

[B28] Camilleri M. Kerstens R. Rykx A. Vandeplassche L. (2008). A Placebo-Controlled Trial of Prucalopride for Severe Chronic Constipation. N. Engl. J. Med. 358 (22), 2344–2354. 10.1056/NEJMoa0800670 18509121

[B29] Camilleri M. (2019). Leaky Gut: Mechanisms, Measurement and Clinical Implications in Humans. Gut 68 (8), 1516–1526. 10.1136/gutjnl-2019-318427 31076401 PMC6790068

[B30] Camilleri M. Lembo A. McCallum R. Tourkodimitris S. Kemps L. Miller M. B. (2020a). Overall Safety of Relamorelin in Adults with Diabetic Gastroparesis: Analysis of Phase 2a and 2b Trial Data. Aliment. Pharmacol. Ther. 51 (11), 1139–1148. 10.1111/apt.15711 32301137 PMC7318559

[B31] Camilleri M. Madsen K. Spiller R. Greenwood-Van Meerveld B. Van Meerveld B. G. Verne G. N. (2012). Intestinal Barrier Function in Health and Gastrointestinal Disease. Neurogastroenterol Motil. 24 (6), 503–512. 10.1111/j.1365-2982.2012.01921.x 22583600 PMC5595063

[B32] Camilleri M. (2021). New Drugs on the Horizon for Functional and Motility Gastrointestinal Disorders. Gastroenterology 161 (3), 761–764. 10.1053/j.gastro.2021.04.079 33989661 PMC8380736

[B33] Camilleri M. Nord S. L. Burton D. Oduyebo I. Zhang Y. Chen J. (2020b). Randomised Clinical Trial: Significant Biochemical and Colonic Transit Effects of the Farnesoid X Receptor Agonist Tropifexor in Patients with Primary Bile Acid Diarrhoea. Aliment. Pharmacol. Ther. 52 (5), 808–820. 10.1111/apt.15967 32702169

[B34] Camilleri M. Sanders K. M. (2022). Gastroparesis. Gastroenterology 162 (1), 68–e1. 10.1053/j.gastro.2021.10.028 34717924 PMC8678360

[B35] Camilleri M. Stanghellini V. (2013). Current Management Strategies and Emerging Treatments for Functional Dyspepsia. Nat. Rev. Gastroenterol. Hepatol. 10 (3), 187–194. 10.1038/nrgastro.2013.11 23381190

[B36] Cani P. D. (2017). Gut Microbiota - at the Intersection of Everything? Nat. Rev. Gastroenterol. Hepatol. 14 (6), 321–322. 10.1038/nrgastro.2017.54 28442782

[B37] Carabotti M. Scirocco A. Maselli M. A. Severi C. (2015). The Gut-Brain axis: Interactions between Enteric Microbiota, central and Enteric Nervous Systems. Ann. Gastroenterol. 28 (2), 203–209. 25830558 PMC4367209

[B38] Carbone F. Van den Houte K. Clevers E. Andrews C. N. Papathanasopoulos A. Holvoet L. (2019). Prucalopride in Gastroparesis: A Randomized Placebo-Controlled Crossover Study. Am. J. Gastroenterol. 114 (8), 1265–1274. 10.14309/ajg.0000000000000304 31295161

[B39] Carlin J. L. Lieberman V. R. Dahal A. Keefe M. S. Xiao C. Birznieks G. (2021). Efficacy and Safety of Tradipitant in Patients with Diabetic and Idiopathic Gastroparesis in a Randomized, Placebo-Controlled Trial. Gastroenterology 160 (1), 76–e4. 10.1053/j.gastro.2020.07.029 32693185

[B40] Castro J. Garcia-Caraballo S. Maddern J. Schober G. Lumsden A. Harrington A. (2021). Olorinab (APD371), a Peripherally Acting, Highly Selective, Full Agonist of the Cannabinoid Receptor 2, Reduces Colitis-Induced Acute and Chronic Visceral Hypersensitivity in Rodents. Pain 163, e72–e86. 10.1097/j.pain.0000000000002314 PMC8675055 33863856

[B41] Charbonneau D. Gibb R. D. Quigley E. M. (2013). Fecal Excretion of Bifidobacterium Infantis 35624 and Changes in Fecal Microbiota after Eight Weeks of Oral Supplementation with Encapsulated Probiotic. Gut Microbes 4 (3), 201–211. 10.4161/gmic.24196 23549409 PMC3669165

[B42] Chedid V. Brandler J. Arndt K. Vijayvargiya P. Wang X. J. Burton D. (2021). Randomised Study: Effects of the 5-HT4 Receptor Agonist Felcisetrag vs Placebo on Gut Transit in Patients with Gastroparesis. Aliment. Pharmacol. Ther. 53 (9), 1010–1020. 10.1111/apt.16304 33711180 PMC8251541

[B43] Chedid V. Camilleri M. (2017). Relamorelin for the Treatment of Gastrointestinal Motility Disorders. Expert Opin. Investig. Drugs 26 (10), 1189–1197. 10.1080/13543784.2017.1373088 28847163

[B44] Chey W. D. Camilleri M. Chang L. Rikner L. Graffner H. (2011). A Randomized Placebo-Controlled Phase IIb Trial of A3309, a Bile Acid Transporter Inhibitor, for Chronic Idiopathic Constipation. Am. J. Gastroenterol. 106 (10), 1803–1812. 10.1038/ajg.2011.162 21606974 PMC3188811

[B45] Chey W. D. Lembo A. J. Lavins B. J. Shiff S. J. Kurtz C. B. Currie M. G. (2012). Linaclotide for Irritable Bowel Syndrome with Constipation: a 26-week, Randomized, Double-Blind, Placebo-Controlled Trial to Evaluate Efficacy and Safety. Am. J. Gastroenterol. 107 (11), 1702–1712. 10.1038/ajg.2012.254 22986437

[B46] Choi K. M. Kashyap P. C. Dutta N. Stoltz G. J. Ordog T. Shea Donohue T. (2010). CD206-positive M2 Macrophages that Express Heme Oxygenase-1 Protect against Diabetic Gastroparesis in Mice. Gastroenterology 138 (7), 23992409e2391–e1. 10.1053/j.gastro.2010.02.014 PMC2883675 20178793

[B47] Cipriani G. Gibbons S. J. Kashyap P. C. Farrugia G. (2016). Intrinsic Gastrointestinal Macrophages: Their Phenotype and Role in Gastrointestinal Motility. Cell Mol Gastroenterol Hepatol 2 (2), 120–e1. e121. 10.1016/j.jcmgh.2016.01.003 27047989 PMC4817106

[B48] Connell M. Shin A. James-Stevenson T. Xu H. Imperiale T. F. Herron J. (2018). Systematic Review and Meta-Analysis: Efficacy of Patented Probiotic, VSL#3, in Irritable Bowel Syndrome. Neurogastroenterol Motil. 30 (12), e13427. 10.1111/nmo.13427 30069978 PMC6249050

[B49] Desai M. S. Seekatz A. M. Koropatkin N. M. Kamada N. Hickey C. A. Wolter M. (2016). A Dietary Fiber-Deprived Gut Microbiota Degrades the Colonic Mucus Barrier and Enhances Pathogen Susceptibility. Cell 167 (5), 1339–e21. e1321. 10.1016/j.cell.2016.10.043 27863247 PMC5131798

[B50] Dothel G. Barbaro M. R. Boudin H. Vasina V. Cremon C. Gargano L. (2015). Nerve Fiber Outgrowth Is Increased in the Intestinal Mucosa of Patients with Irritable Bowel Syndrome. Gastroenterology 148 (5), 1002–e4. e1004. 10.1053/j.gastro.2015.01.042 25655556

[B51] Drossman D. A. Chey W. D. Johanson J. F. Fass R. Scott C. Panas R. (2009). Clinical Trial: Lubiprostone in Patients with Constipation-Associated Irritable Bowel Syndrome-Rresults of Two Randomized, Placebo-Controlled Studies. Aliment. Pharmacol. Ther. 29 (3), 329–341. 10.1111/j.1365-2036.2008.03881.x 19006537

[B52] Drossman D. A. (2016). Functional Gastrointestinal Disorders: History, Pathophysiology, Clinical Features and Rome IV. Gastroenterology S0016-5085 (16), 00223–00227. 10.1053/j.gastro.2016.02.032 27144617

[B53] Drossman D. A. Tack J. Ford A. C. Szigethy E. Törnblom H. Van Oudenhove L. (2018). Neuromodulators for Functional Gastrointestinal Disorders (Disorders of Gut-Brain Interaction): A Rome Foundation Working Team Report. Gastroenterology 154 (4), 1140–e1. e1141. 10.1053/j.gastro.2017.11.279 29274869

[B54] Du L. J. Chen B. R. Kim J. J. Kim S. Shen J. H. Dai N. (2016). *Helicobacter pylori* Eradication Therapy for Functional Dyspepsia: Systematic Review and Meta-Analysis. World J. Gastroenterol. 22 (12), 3486–3495. 10.3748/wjg.v22.i12.3486 27022230 PMC4806206

[B55] Dumitrascu D. L. Weinbeck M. (2000). Domperidone versus Metoclopramide in the Treatment of Diabetic Gastroparesis. Am. J. Gastroenterol. 95 (1), 316–317. 10.1111/j.1572-0241.2000.01721.x 10638616

[B56] El-Salhy M. Hatlebakk J. G. Gilja O. H. Bråthen Kristoffersen A. Hausken T. (2020). Efficacy of Faecal Microbiota Transplantation for Patients with Irritable Bowel Syndrome in a Randomised, Double-Blind, Placebo-Controlled Study. Gut 69 (5), 859–867. 10.1136/gutjnl-2019-319630 31852769 PMC7229896

[B57] El-Salhy M. Patcharatrakul T. Gonlachanvit S. (2021). The Role of Diet in the Pathophysiology and Management of Irritable Bowel Syndrome. Indian J. Gastroenterol. 40 (2), 111–119. 10.1007/s12664-020-01144-6 33666892 PMC8187226

[B58] Enck P. Aziz Q. Barbara G. Farmer A. D. Fukudo S. Mayer E. A. (2016). Irritable Bowel Syndrome. Nat. Rev. Dis. Primers 2, 16014. 10.1038/nrdp.2016.14 27159638 PMC5001845

[B59] Enck P. Azpiroz F. Boeckxstaens G. Elsenbruch S. Feinle-Bisset C. Holtmann G. (2017). Functional Dyspepsia. Nat. Rev. Dis. Primers 3, 17081. 10.1038/nrdp.2017.81 29099093

[B60] Farzaei M. H. Bahramsoltani R. Abdollahi M. Rahimi R. (2016). The Role of Visceral Hypersensitivity in Irritable Bowel Syndrome: Pharmacological Targets and Novel Treatments. J. Neurogastroenterol Motil. 22 (4), 558–574. 10.5056/jnm16001 27431236 PMC5056566

[B61] Ford A. C. Mahadeva S. Carbone M. F. Lacy B. E. Talley N. J. (2020). Functional Dyspepsia. Lancet 396 (10263), 1689–1702. 10.1016/S0140-6736(20)30469-4 33049222

[B62] Ford A. C. Moayyedi P. Lacy B. E. Lembo A. J. Saito Y. A. Schiller L. R. (2014). American College of Gastroenterology Monograph on the Management of Irritable Bowel Syndrome and Chronic Idiopathic Constipation. Am. J. Gastroenterol. 109 (Suppl. 1), S2–S26. 10.1038/ajg.2014.187 25091148

[B63] Fritscher-Ravens A. Schuppan D. Ellrichmann M. Schoch S. Röcken C. Brasch J. (2014). Confocal Endomicroscopy Shows Food-Associated Changes in the Intestinal Mucosa of Patients with Irritable Bowel Syndrome. Gastroenterology 147 (5), 1012–e4. e1014. 10.1053/j.gastro.2014.07.046 25083606

[B64] Gatta L. Scarpignato C. (2017). Systematic Review with Meta-Analysis: Rifaximin Is Effective and Safe for the Treatment of Small Intestine Bacterial Overgrowth. Aliment. Pharmacol. Ther. 45 (5), 604–616. 10.1111/apt.13928 28078798 PMC5299503

[B65] Ghoshal U. C. Bhut B. Misra A. (2021). Patients with Specific Gastrointestinal Motility Disorders Are Commonly Diagnosed as Functional GI Disorders in the Early Stage by Community Physicians Due to Lack of Awareness. Turk J. Gastroenterol. 32 (4), 336–348. 10.5152/tjg.2021.20514 34231480 PMC8975340

[B66] Ghoshal U. C. (2020). Marshall and Warren Lecture 2019: A Paradigm Shift in Pathophysiological Basis of Irritable Bowel Syndrome and its Implication on Treatment. J. Gastroenterol. Hepatol. 35 (5), 712–721. 10.1111/jgh.15032 32162356

[B67] Ghoshal U. C. Singh R. Chang F. Y. Hou X. Wong B. C. Kachintorn U. (2011a). Epidemiology of Uninvestigated and Functional Dyspepsia in Asia: Facts and Fiction. J. Neurogastroenterol Motil. 17 (3), 235–244. 10.5056/jnm.2011.17.3.235 21860815 PMC3155059

[B68] Ghoshal U. C. Singh R. (2017). Frequency and Risk Factors of Functional Gastro-Intestinal Disorders in a Rural Indian Population. J. Gastroenterol. Hepatol. 32 (2), 378–387. 10.1111/jgh.13465 27262283

[B69] Ghoshal U. C. Singh R. (2014). Pathogenesis of Irritable Bowel Syndrome: Is it Really in the Gene? J. Neurogastroenterol Motil. 20 (3), 284–286. 10.5056/jnm14071 24953715 PMC4102161

[B70] Ghoshal U. C. Srivastava D. Misra A. (2018). A Randomized Double-Blind Placebo-Controlled Trial Showing Rifaximin to Improve Constipation by Reducing Methane Production and Accelerating colon Transit: A Pilot Study. Indian J. Gastroenterol. 37 (5), 416–423. 10.1007/s12664-018-0901-6 30406392

[B71] Ghoshal U. C. Srivastava D. Verma A. Misra A. (2011b). Slow Transit Constipation Associated with Excess Methane Production and its Improvement Following Rifaximin Therapy: a Case Report. J. Neurogastroenterol Motil. 17 (2), 185–188. 10.5056/jnm.2011.17.2.185 21602997 PMC3093012

[B72] Gomi A. Yamaji K. Watanabe O. Yoshioka M. Miyazaki K. Iwama Y. (2018). Bifidobacterium Bifidum YIT 10347 Fermented Milk Exerts Beneficial Effects on Gastrointestinal Discomfort and Symptoms in Healthy Adults: A Double-Blind, Randomized, Placebo-Controlled Study. J. Dairy Sci. 101 (6), 4830–4841. 10.3168/jds.2017-13803 29573807

[B73] Gottfried-Blackmore A. Namkoong H. Adler E. Martin B. Gubatan J. Fernandez-Becker N. (2021). Gastric Mucosal Immune Profiling and Dysregulation in Idiopathic Gastroparesis. Clin. Transl Gastroenterol. 12 (5), e00349. 10.14309/ctg.0000000000000349 33979305 PMC8132986

[B74] Grover M. Berumen A. Peters S. Wei T. Breen-Lyles M. Harmsen W. S. (2021). Intestinal Chemosensitivity in Irritable Bowel Syndrome Associates with Small Intestinal TRPV Channel Expression. Aliment. Pharmacol. Ther. 54 (9), 1179–1192. 10.1111/apt.16591 34472640

[B75] Grover M. Farrugia G. Lurken M. S. Bernard C. E. Faussone-Pellegrini M. S. Smyrk T. C. (2011). Cellular Changes in Diabetic and Idiopathic Gastroparesis. Gastroenterology 140 (5), 1575–e8. 10.1053/j.gastro.2011.01.046 21300066 PMC3081914

[B76] Grover M. Farrugia G. Stanghellini V. (2019). Gastroparesis: a Turning point in Understanding and Treatment. Gut 68 (12), 2238–2250. 10.1136/gutjnl-2019-318712 31563877 PMC6874806

[B77] Grover M. Gibbons S. J. Nair A. A. Bernard C. E. Zubair A. S. Eisenman S. T. (2018). Transcriptomic Signatures Reveal Immune Dysregulation in Human Diabetic and Idiopathic Gastroparesis. BMC Med. Genomics 11 (1), 62. 10.1186/s12920-018-0379-1 30086735 PMC6081936

[B78] Guleria A. Karyampudi A. Singh R. Khetrapal C. L. Verma A. Ghoshal U. C. (2017). Mapping of Brain Activations to Rectal Balloon Distension Stimuli in Male Patients with Irritable Bowel Syndrome Using Functional Magnetic Resonance Imaging. J. Neurogastroenterol Motil. 23 (3), 415–427. 10.5056/jnm16148 28192648 PMC5503292

[B79] Herrick L. M. Camilleri M. Schleck C. D. Zinsmeister A. R. Saito Y. A. Talley N. J. (2018). Effects of Amitriptyline and Escitalopram on Sleep and Mood in Patients with Functional Dyspepsia. Clin. Gastroenterol. Hepatol. 16 (3), 401–e2. e402. 10.1016/j.cgh.2017.10.021 29199141

[B80] Ianiro G. Eusebi L. H. Black C. J. Gasbarrini A. Cammarota G. Ford A. C. (2019). Systematic Review with Meta-Analysis: Efficacy of Faecal Microbiota Transplantation for the Treatment of Irritable Bowel Syndrome. Aliment. Pharmacol. Ther. 50 (3), 240–248. 10.1111/apt.15330 31136009

[B81] Iovino P. Bucci C. Tremolaterra F. Santonicola A. Chiarioni G. (2014). Bloating and Functional Gastro-Intestinal Disorders: where Are We and where Are We Going? World J. Gastroenterol. 20 (39), 14407–14419. 10.3748/wjg.v20.i39.14407 25339827 PMC4202369

[B82] Islam B. N. Sharman S. K. Browning D. D. (2018). Clinical Utility of Plecanatide in the Treatment of Chronic Idiopathic Constipation. Int. J. Gen. Med. 11, 323–330. 10.2147/IJGM.S125051 30127634 PMC6089121

[B83] Jacob D. Busciglio I. Burton D. Halawi H. Oduyebo I. Rhoten D. (2017). Effects of NK1 Receptors on Gastric Motor Functions and Satiation in Healthy Humans: Results from a Controlled Trial with the NK1 Antagonist Aprepitant. Am. J. Physiol. Gastrointest. Liver Physiol. 313 (5), G505–G510. 10.1152/ajpgi.00197.2017 28814387 PMC5792217

[B84] Ji S. Traini C. Mischopoulou M. Gibbons S. J. Ligresti G. Faussone-Pellegrini M. S. (2021). Muscularis Macrophages Establish Cell-To-Cell Contacts with telocytes/PDGFRα-Positive Cells and Smooth Muscle Cells in the Human and Mouse Gastrointestinal Tract. Neurogastroenterol Motil. 33 (3), e13993. 10.1111/nmo.13993 33020982 PMC7902307

[B85] Jiang C. Xu Q. Wen X. Sun H. (2015). Current Developments in Pharmacological Therapeutics for Chronic Constipation. Acta Pharm. Sin B 5 (4), 300–309. 10.1016/j.apsb.2015.05.006 26579459 PMC4629408

[B86] Jin B. Ha S. E. Wei L. Singh R. Zogg H. Clemmensen B. (2021). Colonic Motility Is Improved by the Activation of 5-HT2B Receptors on Interstitial Cells of Cajal in Diabetic Mice. Gastroenterology 161 (2), 608–e7. e607. 10.1053/j.gastro.2021.04.040 33895170 PMC8532042

[B87] Johanson J. F. Morton D. Geenen J. Ueno R. (2008). Multicenter, 4-week, Double-Blind, Randomized, Placebo-Controlled Trial of Lubiprostone, a Locally-Acting Type-2 Chloride Channel Activator, in Patients with Chronic Constipation. Am. J. Gastroenterol. 103 (1), 170–177. 10.1111/j.1572-0241.2007.01524.x 17916109

[B88] Johanson J. F. Wald A. Tougas G. Chey W. D. Novick J. S. Lembo A. J. (2004). Effect of Tegaserod in Chronic Constipation: a Randomized, Double-Blind, Controlled Trial. Clin. Gastroenterol. Hepatol. 2 (9), 796–805. 10.1016/s1542-3565(04)00356-8 15354280

[B89] Khanna L. Camilleri M. (2021). Review Article: Elobixibat: a Novel Treatment for Chronic Constipation. Aliment. Pharmacol. Ther. 53 (2), 234–242. 10.1111/apt.16143 33296518

[B90] Khoruts A. Sadowsky M. J. (2016). Understanding the Mechanisms of Faecal Microbiota Transplantation. Nat. Rev. Gastroenterol. Hepatol. 13 (9), 508–516. 10.1038/nrgastro.2016.98 27329806 PMC5909819

[B91] Kim S. E. Park Y. S. Kim N. Kim M. S. Jo H. J. Shin C. M. (2013). Effect of *Helicobacter pylori* Eradication on Functional Dyspepsia. J. Neurogastroenterol Motil. 19 (2), 233–243. 10.5056/jnm.2013.19.2.233 23667755 PMC3644660

[B92] Kim Y. J. Chung W. C. Kim B. W. Kim S. S. Kim J. I. Kim N. J. (2017). Is *Helicobacter pylori* Associated Functional Dyspepsia Correlated with Dysbiosis? J. Neurogastroenterol Motil. 23 (4), 504–516. 10.5056/jnm17066 28992674 PMC5628982

[B93] Klooker T. K. Braak B. Koopman K. E. Welting O. Wouters M. M. van der Heide S. (2010). The Mast Cell Stabiliser Ketotifen Decreases Visceral Hypersensitivity and Improves Intestinal Symptoms in Patients with Irritable Bowel Syndrome. Gut 59 (9), 1213–1221. 10.1136/gut.2010.213108 20650926

[B94] Koga Y. Ohtsu T. Kimura K. Asami Y. (2019). Probiotic L. Gasseri Strain (LG21) for the Upper Gastrointestinal Tract Acting through Improvement of Indigenous Microbiota. BMJ Open Gastroenterol. 6 (1), e000314. 10.1136/bmjgast-2019-000314 PMC6711431 31523442

[B95] Kuo B. Barnes C. N. Nguyen D. D. Shaywitz D. Grimaldi M. Renzulli C. (2021). Velusetrag Accelerates Gastric Emptying in Subjects with Gastroparesis: a Multicentre, Double-Blind, Randomised, Placebo-Controlled, Phase 2 Study. Aliment. Pharmacol. Ther. 53 (10), 1090–1097. 10.1111/apt.16344 33811761

[B96] Lee J. Y. Kim N. Choi Y. J. Park J. H. Ashktorab H. Smoot D. T. (2020). Expression of Tight Junction Proteins According to Functional Dyspepsia Subtype and Sex. J. Neurogastroenterol Motil. 26 (2), 248–258. 10.5056/jnm19208 32235032 PMC7176499

[B97] Malamood M. Roberts A. Kataria R. Parkman H. P. Schey R. (2017). Mirtazapine for Symptom Control in Refractory Gastroparesis. Drug Des. Devel Ther. 11, 1035–1041. 10.2147/DDDT.S125743 PMC5384687 28408802

[B98] Malfertheiner P. MOssner J. Fischbach W. Layer P. Leodolter A. Stolte M. (2003). *Helicobacter pylori* Eradication Is Beneficial in the Treatment of Functional Dyspepsia. Aliment. Pharmacol. Ther. 18 (6), 615–625. 10.1046/j.1365-2036.2003.01695.x 12969088

[B99] Malinen E. Rinttilä T. Kajander K. Mättö J. Kassinen A. Krogius L. (2005). Analysis of the Fecal Microbiota of Irritable Bowel Syndrome Patients and Healthy Controls with Real-Time PCR. Am. J. Gastroenterol. 100 (2), 373–382. 10.1111/j.1572-0241.2005.40312.x 15667495

[B100] Marella H. K. Saleem N. Olden K. (2019). Mirtazapine for Refractory Gastroparesis. ACG Case Rep. J. 6 (10), e00256. 10.14309/crj.0000000000000256 31832475 PMC6855526

[B101] Markham A. (2020). Oliceridine: First Approval. Drugs 80 (16), 1739–1744. 10.1007/s40265-020-01414-9 33025536

[B102] Mars R. A. T. Yang Y. Ward T. Houtti M. Priya S. Lekatz H. R. (2020). Longitudinal Multi-Omics Reveals Subset-specific Mechanisms Underlying Irritable Bowel Syndrome. Cell 182 (6), 1460–e17. e1417. 10.1016/j.cell.2020.08.007 32916129 PMC8109273

[B103] Martínez C. Rodiño-Janeiro B. K. Lobo B. Stanifer M. L. Klaus B. Granzow M. (2017). miR-16 and miR-125b Are Involved in Barrier Function Dysregulation through the Modulation of Claudin-2 and Cingulin Expression in the Jejunum in IBS with Diarrhoea. Gut 66 (9), 1537–1538. 10.1136/gutjnl-2016-311477 PMC5561373 28082316

[B104] Masuy I. Tack J. Verbeke K. Carbone F. (2019a). Acotiamide Affects Antral Motility, but Has No Effect on Fundic Motility, Gastric Emptying or Symptom Perception in Healthy Participants. Neurogastroenterol Motil. 31 (4), e13540. 10.1111/nmo.13540 30663175

[B105] Masuy I. Van Oudenhove L. Tack J. (2019b). Review Article: Treatment Options for Functional Dyspepsia. Aliment. Pharmacol. Ther. 49 (9), 1134–1172. 10.1111/apt.15191 30924176

[B106] Matricon J. Meleine M. Gelot A. Piche T. Dapoigny M. Muller E. (2012). Review Article: Associations between Immune Activation, Intestinal Permeability and the Irritable Bowel Syndrome. Aliment. Pharmacol. Ther. 36 (11-12), 1009–1031. 10.1111/apt.12080 23066886

[B107] Matsueda K. Hongo M. Tack J. Saito Y. Kato H. (2012). A Placebo-Controlled Trial of Acotiamide for Meal-Related Symptoms of Functional Dyspepsia. Gut 61 (6), 821–828. 10.1136/gutjnl-2011-301454 22157329 PMC3345932

[B108] Mazzoleni L. E. Sander G. B. Francesconi C. F. Mazzoleni F. Uchoa D. M. De Bona L. R. (2011). *Helicobacter pylori* Eradication in Functional Dyspepsia: HEROES Trial. Arch. Intern. Med. 171 (21), 1929–1936. 10.1001/archinternmed.2011.533 22123802

[B109] Mazzone A. Strege P. R. Gibbons S. J. Alcaino C. Joshi V. Haak A. J. (2020). microRNA Overexpression in Slow Transit Constipation Leads to Reduced NaV1.5 Current and Altered Smooth Muscle Contractility. Gut 69 (5), 868–876. 10.1136/gutjnl-2019-318747 31757880 PMC7147984

[B110] McColl K. Murray L. El-Omar E. Dickson A. El-Nujumi A. Wirz A. (1998). Symptomatic Benefit from Eradicating *Helicobacter pylori* Infection in Patients with Nonulcer Dyspepsia. N. Engl. J. Med. 339 (26), 1869–1874. 10.1056/NEJM199812243392601 9862941

[B111] Mertz H. Fass R. Kodner A. Yan-Go F. Fullerton S. Mayer E. A. (1998). Effect of Amitriptyline on Symptoms, Sleep, and Visceral Perception in Patients with Functional Dyspepsia. Am. J. Gastroenterol. 93 (2), 160–165. 10.1111/j.1572-0241.1998.00160.x 9468233

[B112] Miwa H. Hirai S. Nagahara A. Murai T. Nishira T. Kikuchi S. (2000). Cure of *Helicobacter pylori* Infection Does Not Improve Symptoms in Non-ulcer Dyspepsia Patients-A Double-Blind Placebo-Controlled Study. Aliment. Pharmacol. Ther. 14 (3), 317–324. 10.1046/j.1365-2036.2000.00706.x 10735925

[B113] Miwa H. Nagahara A. Tominaga K. Yokoyama T. Sawada Y. Inoue K. (2009). Efficacy of the 5-HT1A Agonist Tandospirone Citrate in Improving Symptoms of Patients with Functional Dyspepsia: a Randomized Controlled Trial. Am. J. Gastroenterol. 104 (11), 2779–2787. 10.1038/ajg.2009.427 19638966

[B114] Moayyedi P. Delaney B. C. Vakil N. Forman D. Talley N. J. (2004). The Efficacy of Proton Pump Inhibitors in Nonulcer Dyspepsia: a Systematic Review and Economic Analysis. Gastroenterology 127 (5), 1329–1337. 10.1053/j.gastro.2004.08.026 15521002

[B115] Morgan V. Pickens D. Gautam S. Kessler R. Mertz H. (2005). Amitriptyline Reduces Rectal Pain Related Activation of the Anterior Cingulate Cortex in Patients with Irritable Bowel Syndrome. Gut 54 (5), 601–607. 10.1136/gut.2004.047423 15831901 PMC1774484

[B116] Motta J. P. Wallace J. L. Buret A. G. Deraison C. Vergnolle N. (2021). Gastrointestinal Biofilms in Health and Disease. Nat. Rev. Gastroenterol. Hepatol. 18 (5), 314–334. 10.1038/s41575-020-00397-y 33510461

[B117] Muller P. A. Koscsó B. Rajani G. M. Stevanovic K. Berres M. L. Hashimoto D. (2014). Crosstalk between Muscularis Macrophages and Enteric Neurons Regulates Gastrointestinal Motility. Cell 158 (2), 300–313. 10.1016/j.cell.2014.04.050 25036630 PMC4149228

[B118] Naz F. Malik S. Afzal S. Anwar S. A. (2013). Frequency of Seropositivity of *Helicobacter pylori* in Patients Presenting with Dyspepsia. J. Ayub Med. Coll. Abbottabad 25 (3-4), 50–54. 25226740

[B119] Needham B. D. Kaddurah-Daouk R. Mazmanian S. K. (2020). Gut Microbial Molecules in Behavioural and Neurodegenerative Conditions. Nat. Rev. Neurosci. 21 (12), 717–731. 10.1038/s41583-020-00381-0 33067567

[B120] Pannemans J. Tack J. (2018). How Effective Are Secretagogues for Irritable Bowel Syndrome with Constipation. Gastroenterology 155 (6), 1677–1679. 10.1053/j.gastro.2018.11.005 30419211

[B121] Parkman H. P. Yates K. Hasler W. L. Nguyen L. Pasricha P. J. Snape W. J. (2011). Clinical Features of Idiopathic Gastroparesis Vary with Sex, Body Mass, Symptom Onset, Delay in Gastric Emptying, and Gastroparesis Severity. Gastroenterology 140 (1), 101–115. 10.1053/j.gastro.2010.10.015 20965184 PMC3089423

[B122] Pasricha P. J. Grover M. Yates K. P. Abell T. L. Bernard C. E. Koch K. L. (2021). Functional Dyspepsia and Gastroparesis in Tertiary Care Are Interchangeable Syndromes with Common Clinical and Pathologic Features. Gastroenterology 160 (6), 2006–2017. 10.1053/j.gastro.2021.01.230 33548234 PMC8547190

[B123] Patterson D. Abell T. Rothstein R. Koch K. Barnett J. (1999). A Double-Blind Multicenter Comparison of Domperidone and Metoclopramide in the Treatment of Diabetic Patients with Symptoms of Gastroparesis. Am. J. Gastroenterol. 94 (5), 1230–1234. 10.1111/j.1572-0241.1999.00456.x 10235199

[B124] Pimentel M. Chatterjee S. Chow E. J. Park S. Kong Y. (2006). Neomycin Improves Constipation-Predominant Irritable Bowel Syndrome in a Fashion that Is Dependent on the Presence of Methane Gas: Subanalysis of a Double-Blind Randomized Controlled Study. Dig. Dis. Sci. 51 (8), 1297–1301. 10.1007/s10620-006-9104-6 16832617

[B125] Pimentel M. Lembo A. Chey W. D. Zakko S. Ringel Y. Yu J. (2011). Rifaximin Therapy for Patients with Irritable Bowel Syndrome without Constipation. N. Engl. J. Med. 364 (1), 22–32. 10.1056/NEJMoa1004409 21208106

[B126] Poitras P. Riberdy Poitras M. Plourde V. Boivin M. Verrier P. (2002). Evolution of Visceral Sensitivity in Patients with Irritable Bowel Syndrome. Dig. Dis. Sci. 47 (4), 914–920. 10.1023/a:1014729125428 11991628

[B127] Pulipati P. Sarkar P. Jakkampudi A. Kaila V. Sarkar S. Unnisa M. (2020). The Indian Gut Microbiota-Is it Unique? Indian J. Gastroenterol. 39 (2), 133–140. 10.1007/s12664-020-01037-8 32388710

[B128] Quigley E. M. M. (2017). Microbiota-Brain-Gut Axis and Neurodegenerative Diseases. Curr. Neurol. Neurosci. Rep. 17 (12), 94. 10.1007/s11910-017-0802-6 29039142

[B129] Raehal K. M. Schmid C. L. Groer C. E. Bohn L. M. (2011). Functional Selectivity at the μ-opioid Receptor: Implications for Understanding Opioid Analgesia and Tolerance. Pharmacol. Rev. 63 (4), 1001–1019. 10.1124/pr.111.004598 21873412 PMC3186080

[B130] Rahimi R. Nikfar S. Rezaie A. Abdollahi M. (2009). Efficacy of Tricyclic Antidepressants in Irritable Bowel Syndrome: a Meta-Analysis. World J. Gastroenterol. 15 (13), 1548–1553. 10.3748/wjg.15.1548 19340896 PMC2669938

[B131] Rahman M. M. Ghoshal U. C. Kibria M. G. Sultana N. Yusuf M. A. Nahar S. (2021). Functional Dyspepsia, Peptic Ulcer, and *Helicobacter pylori* Infection in a Rural Community of South Asia: An Endoscopy-Assisted Household Survey. Clin. Transl Gastroenterol. 12 (4), e00334. 10.14309/ctg.0000000000000334 33878048 PMC8052092

[B132] Rao A. S. Camilleri M. (2010). Review Article: Metoclopramide and Tardive Dyskinesia. Aliment. Pharmacol. Ther. 31 (1), 11–19. 10.1111/j.1365-2036.2009.04189.x 19886950

[B133] Rao S. Lembo A. J. Shiff S. J. Lavins B. J. Currie M. G. Jia X. D. (2012). A 12-week, Randomized, Controlled Trial with a 4-week Randomized Withdrawal Period to Evaluate the Efficacy and Safety of Linaclotide in Irritable Bowel Syndrome with Constipation. Am. J. Gastroenterol. 107 (11), 1714–1725. 10.1038/ajg.2012.255 22986440 PMC3504311

[B134] Reigstad C. S. Salmonson C. E. Rainey J. F. 3rd Szurszewski J. H. Linden D. R. Sonnenburg J. L. (2015). Gut Microbes Promote Colonic Serotonin Production through an Effect of Short-Chain Fatty Acids on Enterochromaffin Cells. FASEB J. 29 (4), 1395–1403. 10.1096/fj.14-259598 25550456 PMC4396604

[B135] Rhee S. H. Pothoulakis C. Mayer E. A. (2009). Principles and Clinical Implications of the Brain-Gut-Enteric Microbiota axis. Nat. Rev. Gastroenterol. Hepatol. 6 (5), 306–314. 10.1038/nrgastro.2009.35 19404271 PMC3817714

[B136] Rokkas T. Ekmektzoglou K. Niv Y. (2021). Comparative Effectiveness of 5-hydroxytryptamine 3 Receptor Antagonists in Irritable Bowel Syndrome: a Network Meta-Analysis of Randomized Controlled Studies. Ann. Gastroenterol. 34 (4), 535–546. 10.20524/aog.2021.0619 34276193 PMC8276363

[B137] Sanders K. M. Koh S. D. Ro S. Ward S. M. (2012). Regulation of Gastrointestinal Motility-Iinsights from Smooth Muscle Biology. Nat. Rev. Gastroenterol. Hepatol. 9 (11), 633–645. 10.1038/nrgastro.2012.168 22965426 PMC4793911

[B138] Schroeder B. O. Bäckhed F. (2016). Signals from the Gut Microbiota to Distant Organs in Physiology and Disease. Nat. Med. 22 (10), 1079–1089. 10.1038/nm.4185 27711063

[B139] Sekirov I. Russell S. L. Antunes L. C. Finlay B. B. (2010). Gut Microbiota in Health and Disease. Physiol. Rev. 90 (3), 859–904. 10.1152/physrev.00045.2009 20664075

[B140] Shah E. D. Lacy B. E. Chey W. D. Chang L. Brenner D. M. (2021). Tegaserod for Irritable Bowel Syndrome with Constipation in Women Younger Than 65 Years without Cardiovascular Disease: Pooled Analyses of 4 Controlled Trials. Am. J. Gastroenterol. 116 (8), 1601–1611. 10.14309/ajg.0000000000001313 34047303 PMC8315186

[B141] Shanahan F. Ghosh T. S. O'Toole P. W. (2021). The Healthy Microbiome-What Is the Definition of a Healthy Gut Microbiome? Gastroenterology 160 (2), 483–494. 10.1053/j.gastro.2020.09.057 33253682

[B142] Sharma A. Rao S. S. C. Kearns K. Orleck K. D. Waldman S. A. (2021). Review Article: Diagnosis, Management and Patient Perspectives of the Spectrum of Constipation Disorders. Aliment. Pharmacol. Ther. 53 (12), 1250–1267. 10.1111/apt.16369 33909919 PMC8252518

[B143] Shin A. Preidis G. A. Shulman R. Kashyap P. C. (2019). The Gut Microbiome in Adult and Pediatric Functional Gastrointestinal Disorders. Clin. Gastroenterol. Hepatol. 17 (2), 256–274. 10.1016/j.cgh.2018.08.054 30153517 PMC6314902

[B144] Shukla R. Ghoshal U. Dhole T. N. Ghoshal U. C. (2015). Fecal Microbiota in Patients with Irritable Bowel Syndrome Compared with Healthy Controls Using Real-Time Polymerase Chain Reaction: An Evidence of Dysbiosis. Dig. Dis. Sci. 60 (10), 2953–2962. 10.1007/s10620-015-3607-y 25784074

[B145] Simrén M. Tack J. (2018). New Treatments and Therapeutic Targets for IBS and Other Functional Bowel Disorders. Nat. Rev. Gastroenterol. Hepatol. 15 (10), 589–605. 10.1038/s41575-018-0034-5 29930260

[B146] Singh R. Ghoshal U. C. Kumar S. Mittal B. (2017). Genetic Variants of Immune-Related Genes IL17F and IL10 Are Associated with Functional Dyspepsia: A Case-Control Study. Indian J. Gastroenterol. 36 (5), 343–352. 10.1007/s12664-017-0788-7 28965252

[B147] Singh R. Ha S. E. Wei L. Jin B. Zogg H. Poudrier S. M. (2021a). miR-10b-5p Rescues Diabetes and Gastrointestinal Dysmotility. Gastroenterology 160 (5), 1662–e18. e1618. 10.1053/j.gastro.2020.12.062 33421511 PMC8532043

[B148] Singh R. Mittal B. Ghoshal U. C. (2016). Functional Dyspepsia Is Associated with GNβ3 C825T and CCK-AR T/C Polymorphism. Eur. J. Gastroenterol. Hepatol. 28 (2), 226–232. 10.1097/MEG.0000000000000511 26551933

[B149] Singh R. Wei L. Ghoshal U. C. (2021b). Micro-organic Basis of Functional Gastrointestinal (GI) Disorders: Role of microRNAs in GI Pacemaking Cells. Indian J. Gastroenterol. 40 (2), 102–110. 10.1007/s12664-021-01159-7 33738768

[B150] Singh R. Zogg H. Ro S. (2021c). Role of microRNAs in Disorders of Gut-Brain Interactions: Clinical Insights and Therapeutic Alternatives. Jpm 11 (10), 1021. 10.3390/jpm11101021 34683162 PMC8541612

[B151] Singh R. Zogg H. Wei L. Bartlett A. Ghoshal U. C. Rajender S. (2021d). Gut Microbial Dysbiosis in the Pathogenesis of Gastrointestinal Dysmotility and Metabolic Disorders. J. Neurogastroenterol Motil. 27 (1), 19–34. 10.5056/jnm20149 33166939 PMC7786094

[B152] Singla N. K. Skobieranda F. Soergel D. G. Salamea M. Burt D. A. Demitrack M. A. (2019). APOLLO-2: A Randomized, Placebo and Active-Controlled Phase III Study Investigating Oliceridine (TRV130), a G Protein-Biased Ligand at the μ-Opioid Receptor, for Management of Moderate to Severe Acute Pain Following Abdominoplasty. Pain Pract. 19 (7), 715–731. 10.1111/papr.12801 31162798 PMC6851842

[B153] Spencer N. J. Hu H. (2020). Enteric Nervous System: Sensory Transduction, Neural Circuits and Gastrointestinal Motility. Nat. Rev. Gastroenterol. Hepatol. 17 (6), 338–351. 10.1038/s41575-020-0271-2 32152479 PMC7474470

[B154] Sperber A. D. Bangdiwala S. I. Drossman D. A. Ghoshal U. C. Simren M. Tack J. (2021a). Worldwide Prevalence and Burden of Functional Gastrointestinal Disorders, Results of Rome Foundation Global Study. Gastroenterology 160 (1), 99–e3. e113. 10.1053/j.gastro.2020.04.014 32294476

[B155] Sperber A. D. Freud T. Aziz I. Palsson O. S. Drossman D. A. Dumitrascu D. L. (2021b). Greater Overlap of Rome IV Disorders of Gut-Brain Interactions Leads to Increased Disease Severity and Poorer Quality of Life. Clin. Gastroenterol. Hepatol. S1542-3565 (21), 00580–00582. 10.1016/j.cgh.2021.05.042 34052391

[B156] Sternini C. Anselmi L. Rozengurt E. (2008). Enteroendocrine Cells: a Site of 'taste' in Gastrointestinal Chemosensing. Curr. Opin. Endocrinol. Diabetes Obes. 15 (1), 73–78. 10.1097/MED.0b013e3282f43a73 18185066 PMC2943060

[B157] Sun Q. Jia Q. Song L. Duan L. (2019). Alterations in Fecal Short-Chain Fatty Acids in Patients with Irritable Bowel Syndrome: A Systematic Review and Meta-Analysis. Medicine (Baltimore) 98 (7), e14513. 10.1097/MD.0000000000014513 30762787 PMC6408019

[B158] Suzuki H. Moayyedi P. (2013). *Helicobacter pylori* Infection in Functional Dyspepsia. Nat. Rev. Gastroenterol. Hepatol. 10 (3), 168–174. 10.1038/nrgastro.2013.9 23358394

[B159] Suzuki H. (2017). The Application of the Rome IV Criteria to Functional Esophagogastroduodenal Disorders in Asia. J. Neurogastroenterol Motil. 23 (3), 325–333. 10.5056/jnm17018 28672431 PMC5503281

[B160] Tack J. Janssen P. Masaoka T. Farré R. Van Oudenhove L. (2012). Efficacy of Buspirone, a Fundus-Relaxing Drug, in Patients with Functional Dyspepsia. Clin. Gastroenterol. Hepatol. 10 (11), 1239–1245. 10.1016/j.cgh.2012.06.036 22813445

[B161] Tack J. Ly H. G. Carbone F. Vanheel H. Vanuytsel T. Holvoet L. (2016). Efficacy of Mirtazapine in Patients with Functional Dyspepsia and Weight Loss. Clin. Gastroenterol. Hepatol. 14 (3), 385–e4. 10.1016/j.cgh.2015.09.043 26538208

[B162] Talley N. J. Ford A. C. (2015). Functional Dyspepsia. N. Engl. J. Med. 373 (19), 1853–1863. 10.1056/NEJMra1501505 26535514

[B163] Talley N. J. Janssens J. Lauritsen K. Rácz I. Bolling-Sternevald E. (1999a). Eradication of *Helicobacter pylori* in Functional Dyspepsia: Randomised Double Blind Placebo Controlled Trial with 12 Months' Follow up. The Optimal Regimen Cures Helicobacter Induced Dyspepsia (ORCHID) Study Group. BMJ 318 (7187), 833–837. 10.1136/bmj.318.7187.833 10092259 PMC27795

[B164] Talley N. J. Vakil N. Ballard E. D. Fennerty M. B. Fennerty M. B. (1999b). Absence of Benefit of Eradicating *Helicobacter pylori* in Patients with Nonulcer Dyspepsia. N. Engl. J. Med. 341 (15), 1106–1111. 10.1056/NEJM199910073411502 10511608

[B165] Tan V. P. Liu K. S. Lam F. Y. Hung I. F. Yuen M. F. Leung W. K. (2017). Randomised Clinical Trial: Rifaximin versus Placebo for the Treatment of Functional Dyspepsia. Aliment. Pharmacol. Ther. 45 (6), 767–776. 10.1111/apt.13945 28112426

[B166] Thoua N. M. Murray C. D. Winchester W. J. Roy A. J. Pitcher M. C. Kamm M. A. (2009). Amitriptyline Modifies the Visceral Hypersensitivity Response to Acute Stress in the Irritable Bowel Syndrome. Aliment. Pharmacol. Ther. 29 (5), 552–560. 10.1111/j.1365-2036.2008.03918.x 19076934

[B167] Törnblom H. Drossman D. A. (2015). Centrally Targeted Pharmacotherapy for Chronic Abdominal Pain. Neurogastroenterol Motil. 27 (4), 455–467. 10.1111/nmo.12509 25651186

[B168] Törnblom H. Drossman D. A. (2018). Psychotropics, Antidepressants, and Visceral Analgesics in Functional Gastrointestinal Disorders. Curr. Gastroenterol. Rep. 20 (12), 58. 10.1007/s11894-018-0664-3 30397821 PMC6223713

[B169] Turcotte J. F. Kao D. Mah S. J. Claggett B. Saltzman J. R. Fedorak R. N. (2013). Breaks in the wall: Increased Gaps in the Intestinal Epithelium of Irritable Bowel Syndrome Patients Identified by Confocal Laser Endomicroscopy (With Videos). Gastrointest. Endosc. 77 (4), 624–630. 10.1016/j.gie.2012.11.006 23357497

[B170] Van Oudenhove L. Crowell M. D. Drossman D. A. Halpert A. D. Keefer L. Lackner J. M. (2016). Biopsychosocial Aspects of Functional Gastrointestinal Disorders. Gastroenterology S0016-5085 (16), 1355–1367. 10.1053/j.gastro.2016.02.027 PMC8809487 27144624

[B171] Veldhuyzen van Zanten S. Fedorak R. N. Lambert J. Cohen L. Vanjaka A. (2003). Absence of Symptomatic Benefit of Lansoprazole, Clarithromycin, and Amoxicillin Triple Therapy in Eradication of *Helicobacter pylori* Positive, Functional (Nonulcer) Dyspepsia. Am. J. Gastroenterol. 98 (9), 1963–1969. 10.1111/j.1572-0241.2003.07583.x 14499772

[B172] Vijayvargiya P. Busciglio I. Burton D. Donato L. Lueke A. Camilleri M. (2018). Bile Acid Deficiency in a Subgroup of Patients with Irritable Bowel Syndrome with Constipation Based on Biomarkers in Serum and Fecal Samples. Clin. Gastroenterol. Hepatol. 16 (4), 522–527. 10.1016/j.cgh.2017.06.039 28666948 PMC5745308

[B173] Vijayvargiya P. Camilleri M. (2019). Current Practice in the Diagnosis of Bile Acid Diarrhea. Gastroenterology 156 (5), 1233–1238. 10.1053/j.gastro.2018.11.069 30844373

[B174] Walters J. R. Johnston I. M. Nolan J. D. Vassie C. Pruzanski M. E. Shapiro D. A. (2015). The Response of Patients with Bile Acid Diarrhoea to the Farnesoid X Receptor Agonist Obeticholic Acid. Aliment. Pharmacol. Ther. 41 (1), 54–64. 10.1111/apt.12999 25329562

[B175] Wauters L. Ceulemans M. Frings D. Lambaerts M. Accarie A. Toth J. (2021). Proton Pump Inhibitors Reduce Duodenal Eosinophilia, Mast Cells, and Permeability in Patients with Functional Dyspepsia. Gastroenterology 160 (5), 1521–e9. 10.1053/j.gastro.2020.12.016 33346007

[B176] Wei L. Singh R. Ha S. E. Martin A. M. Jones L. A. Jin B. (2021a). Serotonin Deficiency Is Associated with Delayed Gastric Emptying. Gastroenterology 160 (7), 2451–e19. 10.1053/j.gastro.2021.02.060 33662386 PMC8532026

[B177] Wei L. Singh R. Ro S. Ghoshal U. C. (2021b). Gut Microbiota Dysbiosis in Functional Gastrointestinal Disorders: Underpinning the Symptoms and Pathophysiology. JGH Open 5 (9), 976–987. 10.1002/jgh3.12528 34584964 PMC8454481

[B178] Williams B. B. Van Benschoten A. H. Cimermancic P. Donia M. S. Zimmermann M. Taketani M. (2014). Discovery and Characterization of Gut Microbiota Decarboxylases that Can Produce the Neurotransmitter Tryptamine. Cell Host Microbe 16 (4), 495–503. 10.1016/j.chom.2014.09.001 25263219 PMC4260654

[B179] Wouters M. M. Balemans D. Van Wanrooy S. Dooley J. Cibert-Goton V. Alpizar Y. A. (2016a). Histamine Receptor H1-Mediated Sensitization of TRPV1 Mediates Visceral Hypersensitivity and Symptoms in Patients with Irritable Bowel Syndrome. Gastroenterology 150 (4), 875–e9. 10.1053/j.gastro.2015.12.034 26752109

[B180] Wouters M. M. Vicario M. Santos J. (2016b). The Role of Mast Cells in Functional GI Disorders. Gut 65 (1), 155–168. 10.1136/gutjnl-2015-309151 26194403

[B181] Yoo B. B. Mazmanian S. K. (2017). The Enteric Network: Interactions between the Immune and Nervous Systems of the Gut. Immunity 46 (6), 910–926. 10.1016/j.immuni.2017.05.011 28636959 PMC5551410

[B182] Zhao W. Zhong X. Zhuang X. Ji H. Li X. Li A. (2013). Evaluation of *Helicobacter pylori* Eradication and Drug Therapy in Patients with Functional Dyspepsia. Exp. Ther. Med. 6 (1), 37–44. 10.3892/etm.2013.1109 23935715 PMC3735894

[B183] Zheng H. Liu Y. J. Chen Z. C. Fan G. Q. (2021). miR-222 Regulates Cell Growth, Apoptosis, and Autophagy of Interstitial Cells of Cajal Isolated from Slow Transit Constipation Rats by Targeting C-Kit. Indian J. Gastroenterol. 40 (2), 198–208. 10.1007/s12664-020-01143-7 33792838

[B184] Zikos T. A. Kamal A. N. Neshatian L. Triadafilopoulos G. Clarke J. O. Nandwani M. (2019). High Prevalence of Slow Transit Constipation in Patients with Gastroparesis. J. Neurogastroenterol Motil. 25 (2), 267–275. 10.5056/jnm18206 30870880 PMC6474696

